# Magnetically separable triazine-based Cu(ii)–vitamin B_5_ complex in nitromethane toward efficient heterogeneous cyanation reaction of aryl halides†

**DOI:** 10.1039/d2ra06104j

**Published:** 2023-01-05

**Authors:** Farzaneh Karimi, Masoumeh Jadidi Nejad, Arefe Salamatmanesh, Akbar Heydari

**Affiliations:** a Chemistry Department, Tarbiat Modares University P.O. Box 14155-4838 Tehran Iran heydar_a@modares.ac.ir +98-21-82883455 +98-21-82883444

## Abstract

In the current study, a highly efficient heterogeneous copper catalyst has been developed by supporting copper acetate on a magnetically separable triazine–vitamin B_5_ system. After the successful characterization of the prepared nanoparticles by various techniques such as FT-IR, FE-SEM, EDX/MAP, XRD, TEM, TGA, VSM, and ICP-OES, the catalytic efficiency of them were evaluated in the cyanation reaction of aryl halides in the presence of nitromethane as a non-toxic and cost-effective cyanation source. The cyanation products were obtained in desirable yields. Notably, the magnetic nanocatalyst can be easily recovered and reused at least five times without a significant decrease in its performance.

## Abstract 2

An efficient heterogeneous copper catalyst was developed by supporting copper acetate on magnetically separable triazine–vitamin B_5_. Its catalytic efficiency was evaluated in the cyanation of aryl halides with nitromethane as a non-toxic and cost-effective cyanating source.

## Graphical abstract

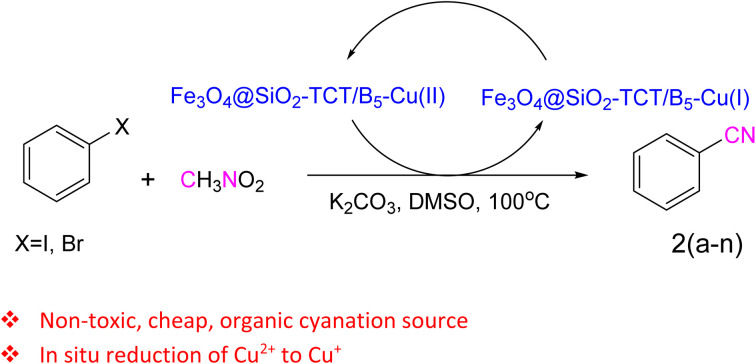

## 1 Introduction

Aromatic nitriles are an important class of versatile scaffolds present in natural compounds and many synthetic organic products, such as dyes, pharmaceuticals, agrochemicals, and herbicides.^1^ They can also be transformed into a variety of organic compounds such as imines, aldehydes, amines, amides, esters, carboxylic acids, and tetrazoles.^2^ In recent decades, novel methodologies have been developed for the introduction of a nitrile functional group into an aromatic framework. The diazotization of anilines followed by the Sandmeyer reaction^3^ and also the Rosenmund–von Braun reaction^4^ have been known as classic synthetic methods toward aryl nitriles. Since then, a series of transition metal-catalyzed protocols have been reported for the cyanation reactions by using diverse cyano sources such as KCN,^5^ NaCN,^6^ Zn(CN)_2_,^7^ CuCN,^8^ and TMSCN.^9^ Nevertheless, most of them are confronted with significant drawbacks including the use of toxic metal cyanides in stoichiometric amounts and the requirement of harsh reaction conditions. To overcome this problems, several less-toxic metal-free cyanide sources including malononitrile,^10^ butyronitrile,^11^ acetonitrile,^12^ benzyl cyanide,^13^ AIBN,^14^*etc.* have been disclosed. Furthermore, some other organic compounds, which can generate cyano groups *in situ* have been employed as indirect cyanation sources. Explored examples of such cyanation reagents include NH_4_I/DMF,^15^ NH_4_HCO_3_/DMSO,^16^ NH_4_HCO_3_/DMF,^17^*t*-BuNC,^18^*etc.* Recently, nitromethane has also been introduced as a potentially valuable indirect organic cyano source for the cyanation of organic scaffolds, because of its chemical stability, and low-cost commercial availability. Nevertheless, there are very few reports on the use of nitromethane as a cyanation reagent in the field of metal-catalyzed aromatic CCN bond formation. So, it is still demanding to make attempts in this direction.^19^

Over the recent past decades, various transition metals, such as Pd,^20^ Co,^21^ Ni,^22^ Cu,^10^ Rh^23^ and Zn^24^ have been utilized to catalyze cyanation reactions of aryls and aryl halides using different cyanation sources. Among them, the use of Cu as a readily available and inexpensive transition metal can be desirable.^17,19^ However, most of these reported metal-catalyzed cyanation reactions have adopted a homogeneous approach, which suffer from serious limitations including high expenses, problematic separation (in some cases non-separable), and non-reusability of metal catalysts. To meet these restrictions as well as minimize possible pollution caused by toxic metal discharge in some homogeneous protocols, a wide range of heterogeneous metal-incorporating catalytic systems have been developed for cyanation transformations.^25^ Magnetic nanoparticles have been well known as one of the most promising heterogeneous nanostructures, which have unique physicochemical features in addition to being easily recoverable and reusable.^26^

Until now, various reports have been presented about the use of ligands in the cyanidation reaction, and by examining the used ligands and the importance of ligands containing nitrogen and oxygen in binding to various metals and forming stable complexes,^27–30,19*a*^ we chose vitamin B_5_ as a suitable ligand for this reaction.

To the best of our knowledge, no heterogeneous copper catalyst has been reported for the cyanation of aryl halides using nitromethane as an indirect organic nitrile source. In this work, we designed and synthesized a novel heterogeneous copper-incorporating nanocatalyst by loading copper acetate on a magnetically recyclable triazine–vitamin B_5_ system for the synthesis of aryl nitriles from aryl halide substrates in presence of nitromethane.

## 2 Experimental

### 2.1. General remarks

All required materials were purchased from Aldrich and Merck companies and were used without any further purification. The reaction was monitored by thin-layer chromatography. TLC was performed on glass plates incorporated with silica-gel 60 F-254. Infrared spectrums (IR) were determined by grade KBr on a Nicolet FT-IR 100 spectrometer. X-ray diffraction (XRD) data were obtained at room temperature by a Philips X-pert 1710. The size and morphology of the nanoparticles were determined by scanning electron microscopy (SEM) using a TESCAN MIRA III FE-SEM. Also, the elemental composition of the nanoparticles was studied by energy-dispersive X-ray (EDAX). Transmission electron microscopy (TEM) was performed using a Philips CM 120 at 120 kV. Thermal gravimetric analysis (TGA) was performed by a thermal analyzer with a heating rate of 10 °C min^−1^ in the range of 25–800 °C under the following air.

### 2.2. Synthesis of TCT/B_5_

Cyanuric chloride (1 mmol or 0.18 g) was dissolved in 5 mL of anhydrous tetrahydrofuran (THF). The resulting solution was then slowly added to a solution of vitamin B_5_ in anhydrous THF (5 mL) and ultrasonicated at 60 °C. Potassium carbonate (1 mmol or 0.138 g) was added to the reaction mixture, and the solution was kept under the same conditions for 3 h and then stirred for 12 h at 70 °C. Finally, the reaction mixture was centrifuged, and the product was separated, washed with anhydrous THF, and dried in a vacuum oven at 70 °C.

### 2.3. Synthesis of Fe_3_O_4_@SiO_2_

A mixture of FeCl_3_·6H_2_O (5 mmol or 1.35 g) and FeCl_2_·4H_2_O (2.5 mmol or 0.5 g) in 50 mL deionized water was ultrasonicated for 10 min to be completely dispersed. While the solution was being stirred at room temperature, 20 mL of ammonia solution (37%) was added dropwise until reaching pH = 11. The solution was then stirred under a nitrogen atmosphere at 80 °C for 5 h. Thereafter, the solution was cooled down to 40 °C, 40 mL of ethanol was added, and the reaction mixture was stirred for 30 min. Tetraethyl orthosilicate (10 mL) was gradually added to the reaction mixture and it was then placed under a nitrogen atmosphere at 40 °C for 22 h. The Fe_3_O_4_@SiO_2_ product was separated by applying an external magnetic field, washed several times with water and ethanol, and dried in a vacuum oven at 80 °C.

### 2.4. Synthesis of Fe_3_O_4_@SiO_2_–TCT/B_5_

Anhydrous toluene (50 mL) was added to 0.2 g Fe_3_O_4_@SiO_2_, and the solution was ultrasonicated for 15 min to be completely dispersed. Then, (3-chloropropyl)trimethoxysilane (10 mL) was added to the dispersion, and the mixture was stirred at 60 °C for 18 h under a nitrogen atmosphere. The nanoparticles were separated by applying an external magnetic field, washed several times with anhydrous toluene, and dried in a vacuum oven at 80 °C. First, potassium iodide (2 mmol or 0.332 g), then potassium carbonate (2 mmol or 0.276 g), and, finally, TA/B_5_ (0.2 g) were added to Fe_3_O_4_@SiO_2_–Cl (0.2 g) dispersed in 10 mL acetonitrile, and the mixture was refluxed for 12 h. The nanoparticles were then separated by applying an external magnetic field, washed several times with water and ethanol, and dried in a vacuum oven at 80 °C.

### 2.5. Synthesis of Fe_3_O_4_@SiO_2_–TCT/B_5_–Cu(ii)

Fe_3_O_4_@SiO_2_–TCT/B_5_ (0.2 g) was dispersed in 50 mL ethanol through 15 min ultrasonication. Next, 1 mmol of copper(ii) acetate monohydrate dissolved in ethanol was added dropwise to the dispersion. The resulting mixture was then refluxed for 16 h. The nanoparticles are separated by applying an external electric field, washed several times with ethanol, and dried in a vacuum oven at 60 °C.

### 2.6. Procedure route for the synthesis of aryl cyanides

First, 4-iodoanisole (1 mmol or 0.234 g), nitromethane (3 mmol or 0.183 g), potassium carbonate (1 mmol or 0.138 g), and the catalyst (30 mg) were weighed and poured into a glass tube. Then, 3 mL dimethyl sulfoxide (solvent) was added and the mixture was stirred at 100 °C for 12 h. Thin-layer chromatography was used to monitor the progress of the reaction. Upon the completion of the reaction, the reaction mixture was cooled down to room temperature, and the catalyst was removed by a magnet. The reaction mixture was then extracted by water and ethyl acetate. After any remaining water droplets were removed from the organic phase by using anhydrous sodium sulfate, the solvent was evaporated under a vacuum. Finally, the purification process was carried out using silica gel plates and ethyl acetate : hexane (1 : 9) solvent.

## 3 Results and discussion

### 3.1. Catalyst preparation

**Scheme 1:**
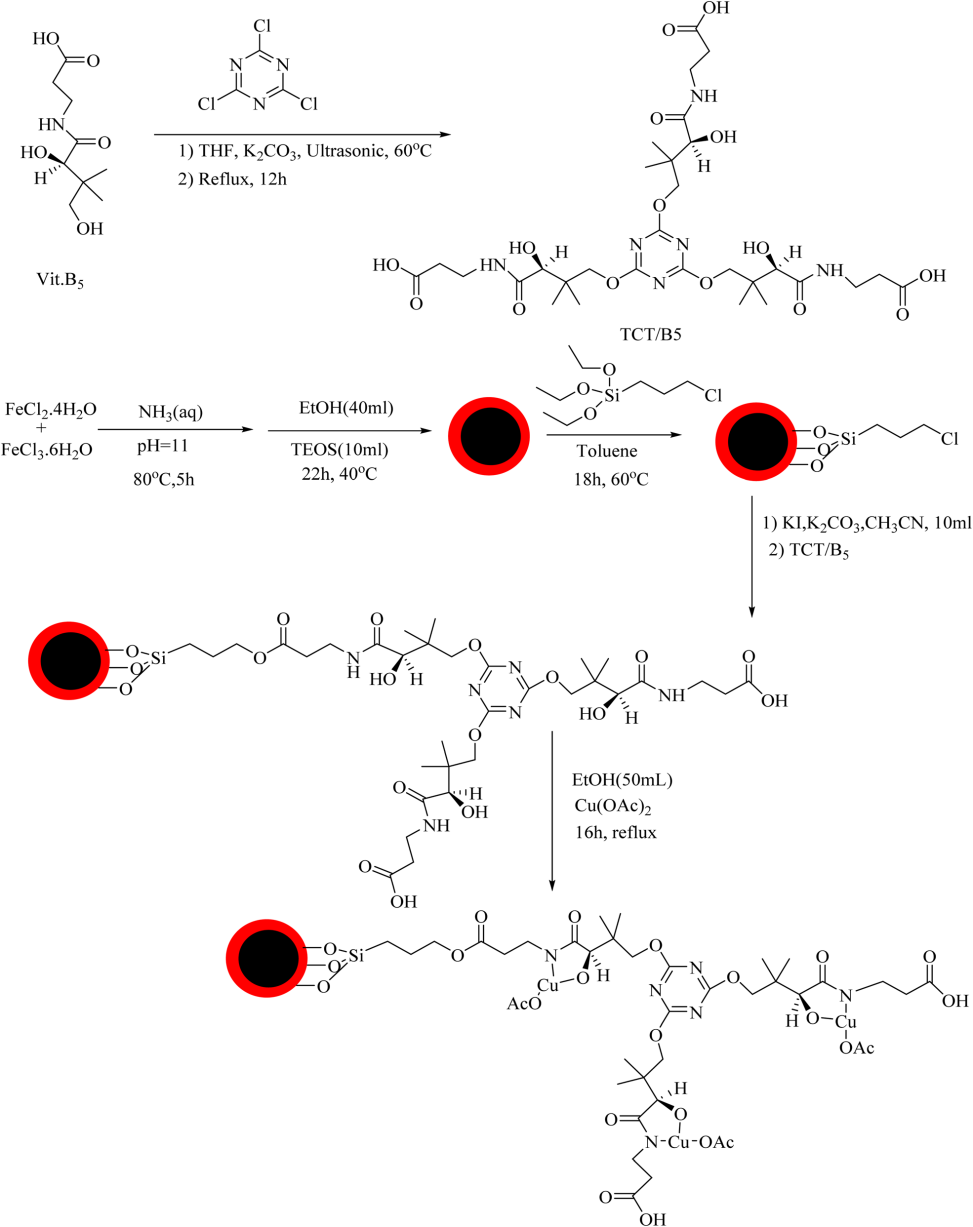
Preparation of Fe_3_O_4_@SiO_2_–TCT/B_5_–Cu(ii).

The preparation process of the proposed Fe_3_O_4_@SiO_2_–TCT/B_5_–Cu(ii) nanocatalyst is illustrated in Scheme 1 as explained in the experimental section. Then to elucidate the structure of the prepared nanocatalyst, several analyses including FT-IR, XRD, SEM, TEM, EDX, TGA, and ICP were carried out.

### 3.2. Catalyst characterization

#### 3.2.1. FT-IR

**Fig. 1:**
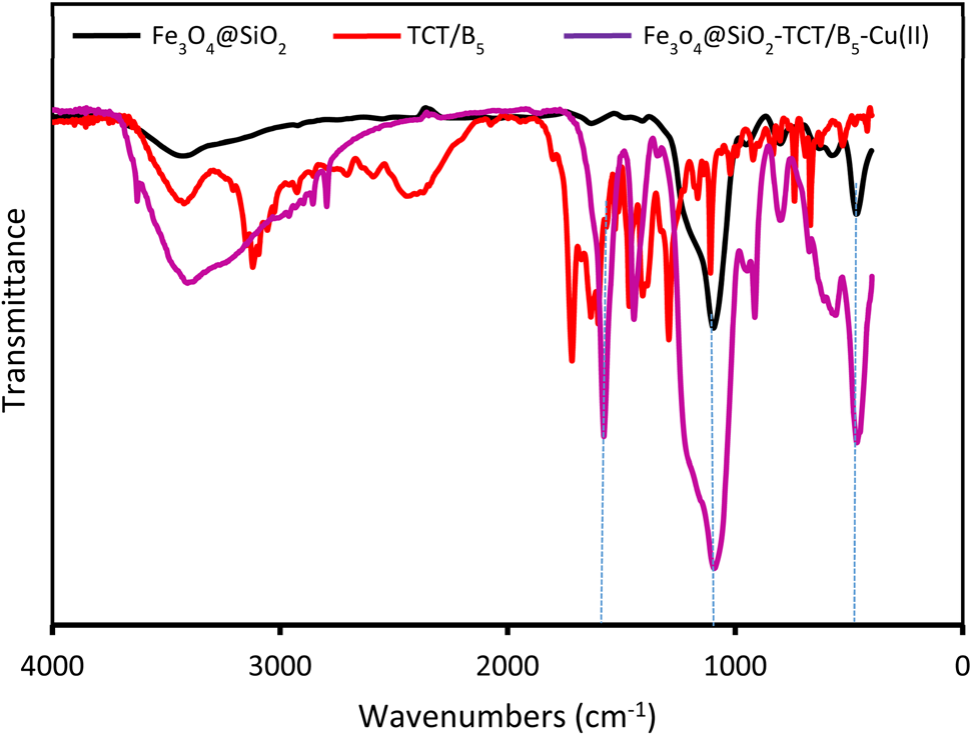
The FT-IR spectra of Fe_3_O_4_@SiO_2_ (black curve), TCT/B_5_ (red curve), and Fe_3_O_4_@SiO_2_–TCT/B_5_–Cu(ii) (violet curve).

FT-IR spectra of Fe_3_O_4_@SiO_2_, TCT/B_5_, and Fe_3_O_4_@SiO_2_–TCT/B_5_–Cu(ii) are shown in Fig. 1. In the spectrum of Fe_3_O_4_@SiO_2_, the stretching and bending vibrations of the O–H bond are observed at around 3429 cm^−1^ and 1632 cm^−1^, respectively. Also, peaks appearing at 1095 cm^−1^ and 466 cm^−1^ are associated with the stretching and bending vibrational modes of Si–O–Si, respectively. In addition, the characteristic peak that appeared at 574 cm^−1^ can be related to the Fe–O stretching. In the FT-IR spectrum of TCT/B_5_, the bands located at 1717 cm^−1^, 1634, cm^−1^ and 1602 cm^−1^ can be assigned to ester, acid and amid carbonyl groups, respectively. The appearance of a characteristic peak at 1717 cm^−1^ can be ascribed to ester, indicating the formation of the covalent bond between TCT and vitamin B_5_. There are two absorption bands in the area of 1400–1460 cm^−1^ and 1100–1290 cm^−1^, which are associated with the stretching vibrations of the C–N bond and C–O bond, respectively. Also, peaks observed at around 3422 cm^−1^ and 2925 cm^−1^ are allocated to O–H and C–H aliphatic stretching vibrations, respectively. The characteristic peaks in the FT-IR spectrum of Fe_3_O_4_@SiO_2_–TCT/B_5_–Cu(ii) are mainly observed at 3406 cm^−1^, 1577 cm^−1^, 1440 cm^−1^, 1092 cm^−1^, and 465 cm^−1^, which can be respectively related to the stretching vibrations of O–H, C=N triazine ring, C–N, Si–O–Si and Fe–O bonds, indicating the successful attachment of TCT/B_5_ to the support. In addition, due to the coordination of some ligand groups with Cu(ii) species, the intensity of some TCT/B_5_ peaks in the final catalyst has decreased.

#### 3.2.2. XRD

**Fig. 2:**
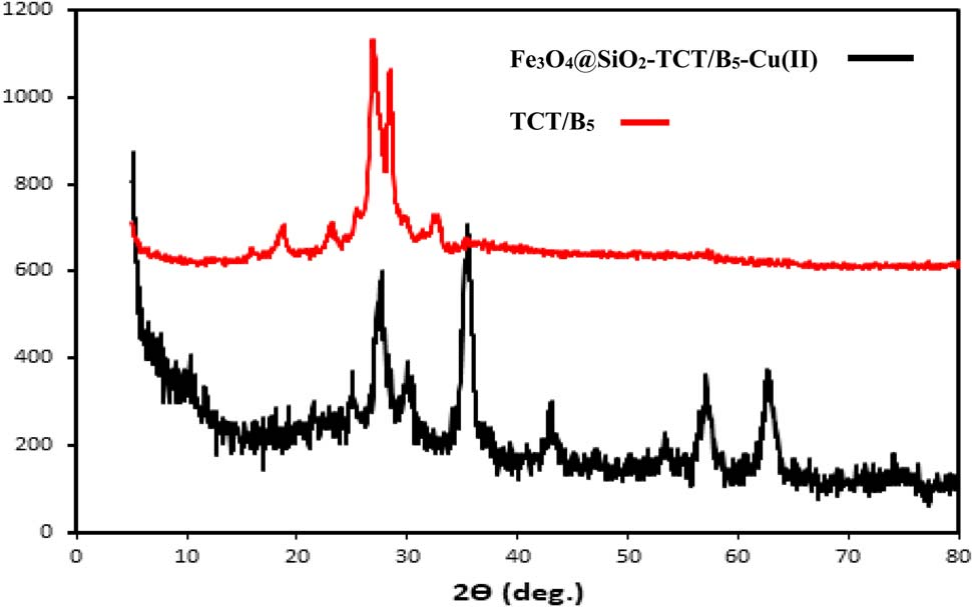
XRD patterns of TCT/B_5_ (red curve) and Fe_3_O_4_@SiO_2_–TCT/B_5_–Cu(ii) (black curve).

XRD results of TCT/B_5_ and Fe_3_O_4_@SiO_2_–TCT/B_5_–Cu(ii) were depicted in Fig. 2. As shown in the XRD pattern of TCT/B_5_, there are peaks to those of ligand structure in the final catalyst XRD pattern. As illustrated in the XRD pattern of Fe_3_O_4_@SiO_2_–TCT/B_5_–Cu(ii), the diffraction peaks at 2*θ* values of 30.74°, 35.89°, 43.44°, 53.94°, 57.64° and 63.24° correspond to (220), (311), (400), (422), (422), (511) and (440) planes of Fe_3_O_4_, which can be readily indexed to the standard spinel cubic magnetite (JCPDS no. 19-0629). Also, the broad peak at 2*θ* = 20 to 30 can be assigned to the amorphous SiO_2_ phase. The diffraction peaks at 2*θ* values of 29.54° and 32.19° can be attributed to ligand structure in the final catalyst. As shown in the XRD pattern of TCT/B_5_, there are peaks similar to those of ligand structure in the final catalyst XRD pattern, confirming the success of ligand binding to the magnetic support.

#### 3.2.3. SEM

**Fig. 3:**
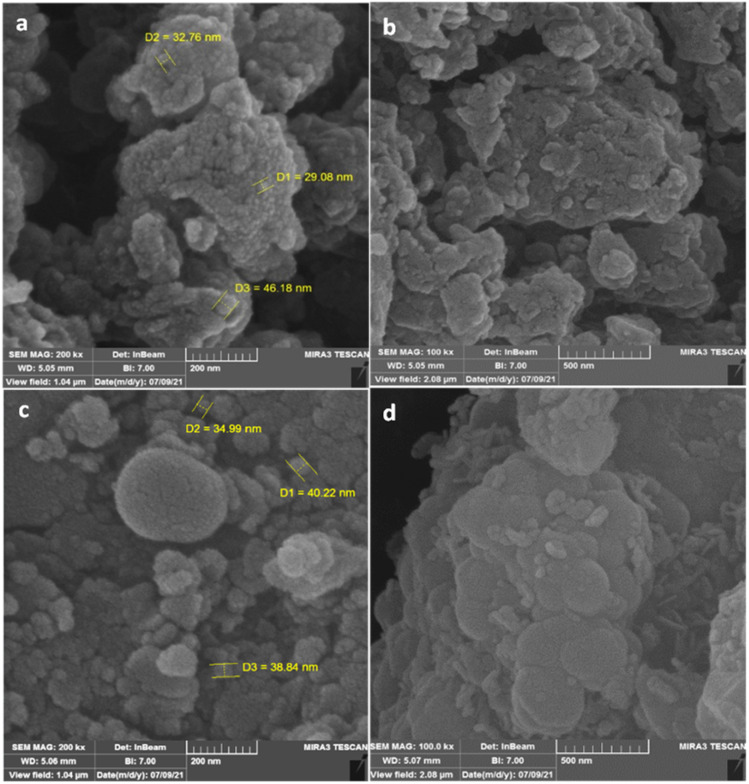
The SEM images of TCT/B_5_ (a and b) and Fe_3_O_4_@SiO_2_–TCT/B_5_–Cu(ii) (c and d).

The morphology and size of nanocatalysts were investigated by FE-SEM analysis. The SEM images of TCT/B_5_ and Fe_3_O_4_@SiO_2_–TCT/B_5_–Cu(ii) were depicted in Fig. 3a–d. As it can be shown, TCT/B_5_ nanoparticles are porous and irregular in shape. The TCT/B_5_ nanoparticles have mean diameter of 36 nm. The morphology of the prepared Fe_3_O_4_@SiO_2_–TCT/B_5_–Cu(ii) nanoparticles is a combination of the spherical Fe_3_O_4_@SiO_2_ nanoparticles with the porous and irregular structure of the TCT/B_5_ ligand with mean diameter of 38 nm, which is well showed in Fig. 3c and d.

#### 3.2.4. EDAX

**Fig. 4:**
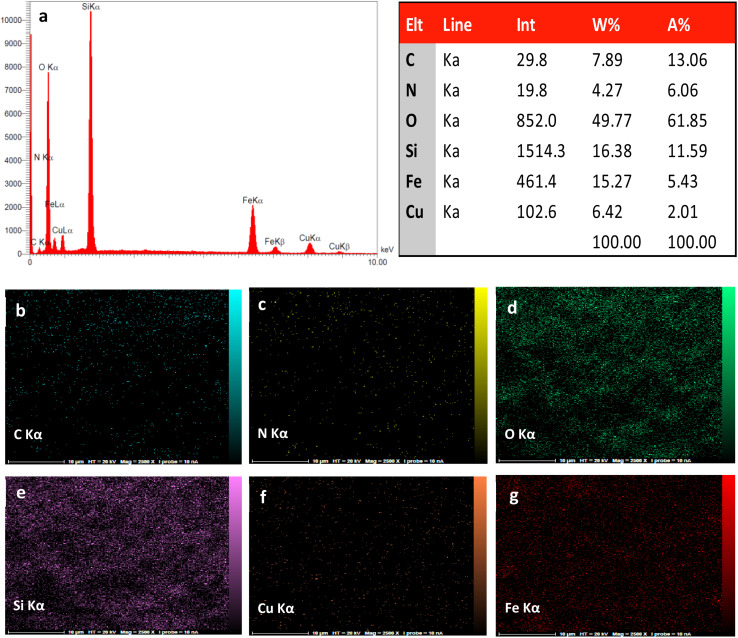
EDAX analysis of Fe_3_O_4_@SiO_2_–TCT/B_5_–Cu(ii) (a) and element-mapping images of Fe_3_O_4_@SiO_2_–TCT/B_5_–Cu(ii) (b–g).

EDAX analysis confirmed that the final catalyst contains the expected elements of C, O, N, Fe, Si, and Cu with wt% of 7.89, 49.77, 4.27, 15.27, 16.38 and 6.42, respectively. Additionally, from EDAX element mapping images (Fig. 4b–h) can be also concluded that TCT/B_5_ and Cu(ii) have a uniform distribution on the surface of Fe_3_O_4_@SiO_2_.

#### 3.2.5. TEM

**Fig. 5:**
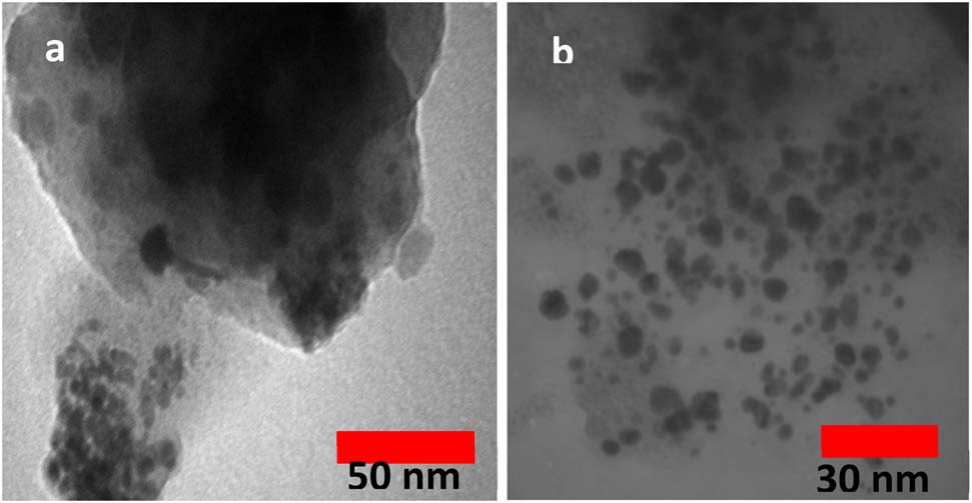
TEM images of Fe_3_O_4_@SiO_2_–TCT/B_5_–Cu(ii) (a and b).

The TEM images of Fe_3_O_4_@SiO_2_–TCT/B_5_–Cu(ii) have been presented in Fig. 5.There are two different morphologies in TEM images of Fe_3_O_4_@SiO_2_–TCT/B_5_–Cu(ii), which can be attributed to the ligand and Fe_3_O_4_ or Cu(ii) NPs. The magnetic core can be seen as a dark spot inside the bright, spherical Cu(ii) triazine-based complex, according to the observation from the TEM images.

#### 3.2.6. TGA

**Fig. 6:**
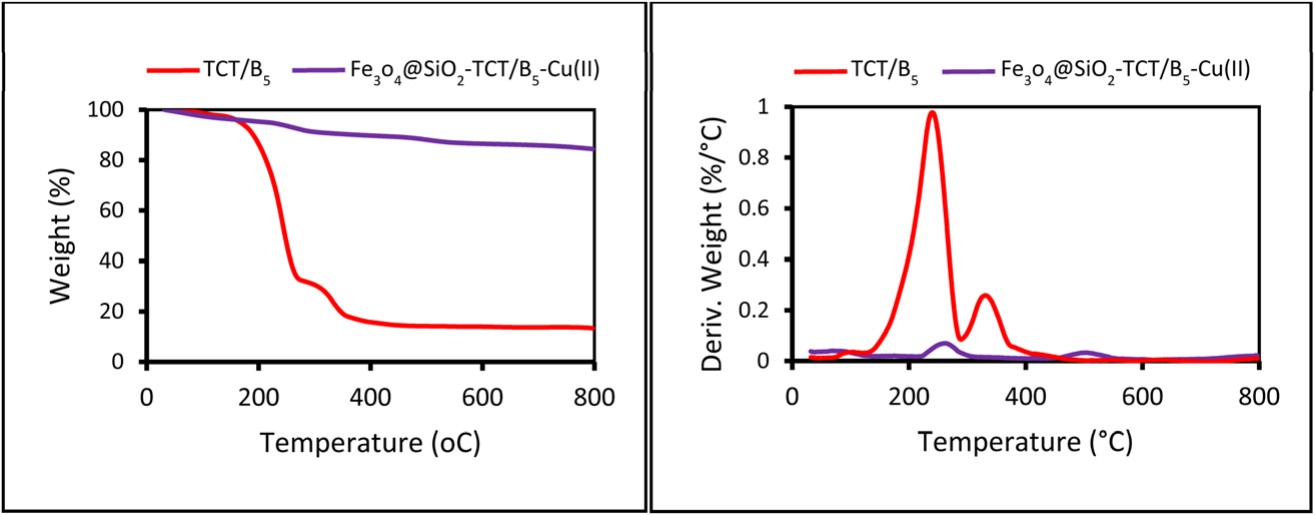
TGA-DTG of TCT/B_5_ (red curve) and Fe_3_O_4_@SiO_2_–TCT/B_5_–Cu(ii) (violet curve).

The thermal degradation and thermal stability of TCT/B_5_ and Fe_3_O_4_@SiO_2_–TCT/B_5_–Cu(ii) nanoparticles were estimated by TGA-DTG analysis. The TGA of TCT/B_5_ (Fig. 6) showed two main weight losses in the range of 150–800 °C. When the temperature increased from 150 to 300 °C, the pyrolysis of TCT/B_5_ particles occurred with a mass loss of about 65%, which can be assigned to the degradation of main chains and aromatic rings. The second weight loss of about 15% between 300–450 °C can be related to the oxidation of carbon. As shown in the TGA-DTG of Fe_3_O_4_@SiO_2_–TCT/B_5_–Cu(ii), the prepared nanoparticles showed higher stability than TCT/B_5_. The first main weight loss of Fe_3_O_4_@SiO_2_–TCT/B_5_–Cu(ii) (about 6%) was observed in the range of 220–340 °C, indicating the degradation of organic contents. The second one (mass loss of 5%) was observed in the range of 460–550 °C, which can be attributed to the decomposition of the remaining organic moieties and oxidation of carbon.

### 3.3. Application of Fe_3_O_4_@SiO_2_–TCT/B_5_–Cu(ii) catalyst in cyanation reaction

**Scheme 2:**
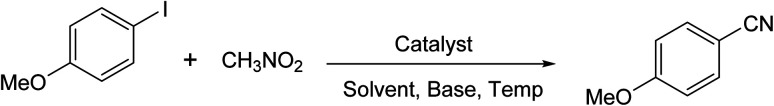
The model reaction for cyanation of aryl halides.

After characterization of the Fe_3_O_4_@SiO_2_–TCT/B_5_–Cu(ii) catalyst, we studied its catalytic performance in the cyanation reaction of aryl halides using CH_3_NO_2_ as the cyanide resource (Scheme 2). In order to optimize the reaction conditions, we selected the cyanation of 4-iodoanisole with CH_3_NO_2_ as the model reaction (Table 1). First, the cyanation reaction was evaluated in the absence of a catalyst (entry 1), no product was obtained under this condition, confirming the need for a catalyst to afford the desired product. The reaction was also tested in the presence of Fe_3_O_4_@SiO_2_–TCT/B_5_ as a catalyst and no product was detected, indicating the necessity of copper for the formation an ideal product. The impacts of solvent, base, temperature, and amount of catalyst were studied to improve the reaction yield. To investigate the role of solvent in the cyanation reaction, a variety of solvents were applied in the reaction (entries 4–9). DMSO was chosen as the best solvent. Comparison between different bases used (entries 10–13), exhibited that K_2_CO_3_ has the best efficiency to form the cyanated product. Based on the catalyst loading study, various amounts of catalyst were tested in this reaction. The amount of 30 mg was found to be the best value of catalyst for high conversion under the reaction conditions. The model reaction in presence of lower values of the catalyst resulted in a decrease in the yield of the cyanation product (entries 14 and 15). Decreasing the reaction temperature to 90 °C brought down the yield to 81% (entry 18). By increasing the temperature to 110 °C (entry 17), no change in the yield of the product was observed. The cyanation reaction was carried out in the presence of copper on substrates Fe_3_O_4_ and Fe_3_O_4_@SiO_2_. These substrates are free of vitamin B_5_ to investigate the importance of the ligand in this reaction. According to the results, the importance of vitamin B_5_ in the prepared composite is concluded. The presence of vitamin B_5_ in the structure of catalyst is essential and enhances efficiency (entries 20 and 21).

**Table 1:** Optimization conditions for Fe_3_O_4_@SiO_2_–TCT/B_5_–Cu(ii)-catalyzed cyanation reaction^a^

Entry	Catalyst	Catalyst (mg)	Base	Solvent	Temp. (°C)	Yield^b^ (%)
1	—	—	K_2_CO_3_	DMSO	100	0
2	Cu(OAc)_2_	10 mol%	K_2_CO_3_	DMSO	100	38
3	Fe_3_O_4_@SiO_2_–TCT/B_5_	30	K_2_CO_3_	DMSO	100	0
4	Fe_3_O_4_@SiO_2_–TCT/B_5_–Cu(ii)	30	K_2_CO_3_	DMSO	100	94
5	Fe_3_O_4_@SiO_2_–TCT/B_5_–Cu(ii)	30	K_2_CO_3_	K_2_CO_3_/glycerol (1 : 5)	100	10
6	Fe_3_O_4_@SiO_2_–TCT/B_5_–Cu(ii)	30	K_2_CO_3_	CH_3_OH	100	70
7	Fe_3_O_4_@SiO_2_–TCT/B_5_–Cu(ii)	30	K_2_CO_3_	Toluene	100	65
8	Fe_3_O_4_@SiO_2_–TCT/B_5_–Cu(ii)	30	K_2_CO_3_	DMF	100	85
9	Fe_3_O_4_@SiO_2_–TCT/B_5_–Cu(ii)	30	K_2_CO_3_	CH_3_CN	100	76
10	Fe_3_O_4_@SiO_2_–TCT/B_5_–Cu(ii)	30	NaHCO_3_	DMSO	100	51
11	Fe_3_O_4_@SiO_2_–TCT/B_5_–Cu(ii)	30	NaOH	DMSO	100	82
12	Fe_3_O_4_@SiO_2_–TCT/B_5_–Cu(ii)	30	(CH_3_)_2_HN	DMSO	100	59
13	Fe_3_O_4_@SiO_2_–TCT/B_5_–Cu(ii)	30	DABCO	DMSO	100	68
14	Fe_3_O_4_@SiO_2_–TCT/B_5_–Cu(ii)	20	K_2_CO_3_	DMSO	100	84
15	Fe_3_O_4_@SiO_2_–TCT/B_5_–Cu(ii)	10	K_2_CO_3_	DMSO	100	71
16	Fe_3_O_4_@SiO_2_–TCT/B_5_–Cu(ii)	40	K_2_CO_3_	DMSO	100	94
17	Fe_3_O_4_@SiO_2_–TCT/B_5_–Cu(ii)	30	K_2_CO_3_	DMSO	110	94
18	Fe_3_O_4_@SiO_2_–TCT/B_5_–Cu(ii)	30	K_2_CO_3_	DMSO	90	81
19	Fe_3_O_4_@SiO_2_–TCT/B_5_–Cu(ii)	30	K_2_CO_3_	DMSO	100	95^c^
20	Fe_3_O_4_/Cu(ii)	30	K_2_CO_3_	DMSO	100	33
21	Fe_3_O_4_@SiO_2_–Cu(ii)	30	K_2_CO_3_	DMSO	100	39

a Reaction conditions: 4-iodoanisole (1 mmol or 0.234 g), nitromethane (3 mmol or 0.183 g), base (1 mmol), and catalyst (30 mg, 0.3 mol%), 12 h, 100 °C.

b Isolated yield.

c K_2_CO_3_ (2 mmol).

In order to evaluate the role of substituent groups, various aryl halides bearing both electron-rich and electron-poor groups were tested in this reaction (Table 2). Under the optimum reaction conditions, aryl iodides and bromides containing various substituents smoothly underwent the cyanation reaction to generate the corresponding benzonitriles in good to excellent yields. Aryl iodides provided higher reaction efficiency than aryl bromides due to the weakness of the C–I bond compared to C–Br. As shown in Table 2, *O*-substituted aryl halides (2f, 2k and 2l) gave lower yields compared to *P*-substitutes due to the hindrance effect.

**Table 2:** Synthesis of benzonitrile derivatives by Fe_3_O_4_@SiO_2_–TCT/B_5_–Cu(ii)^a^

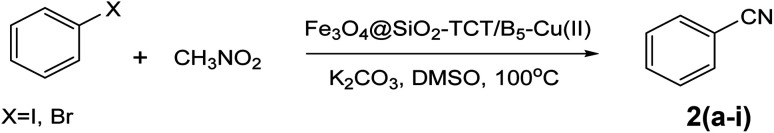			
Entry	Substrate	Product	Yield^b^ (%)
1	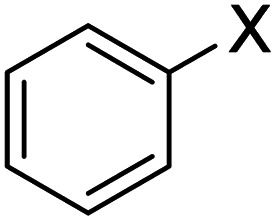	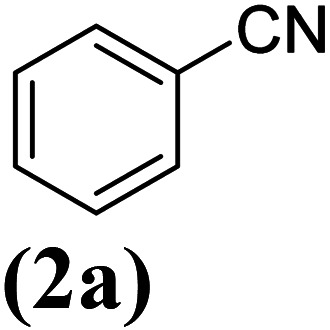	X = I, 61
			X = Br, 45
2	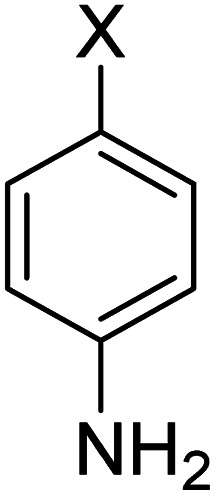	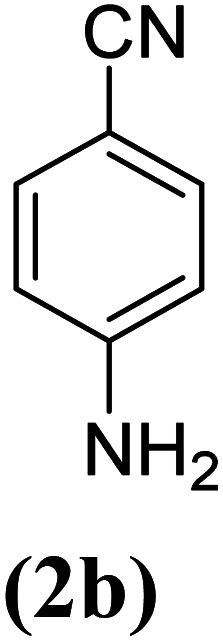	X = I, 80
			X = Br, 73
3	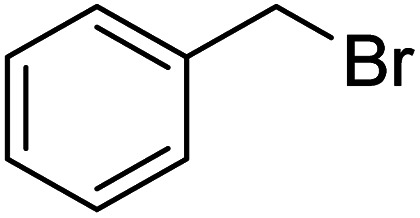	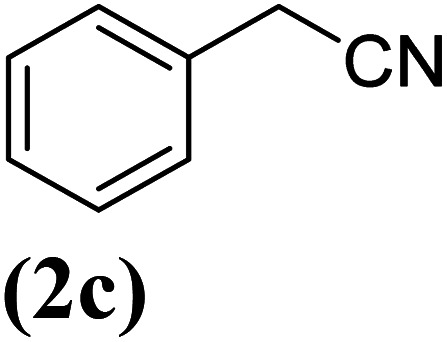	67
4	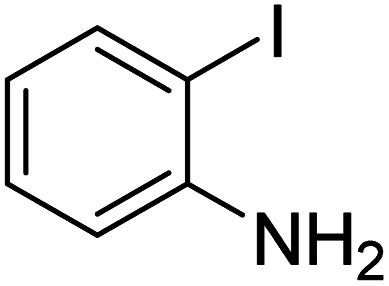	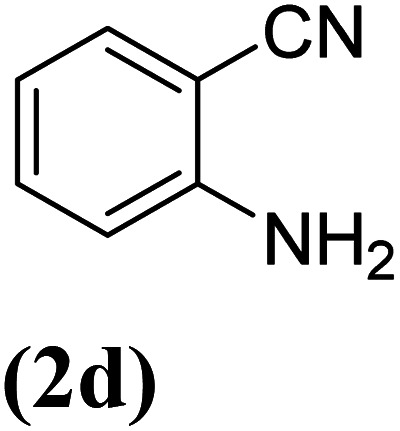	63
5	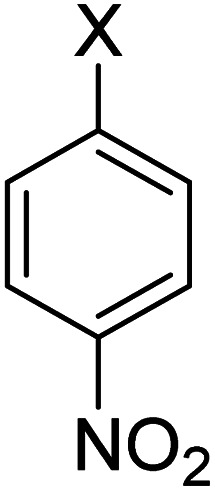	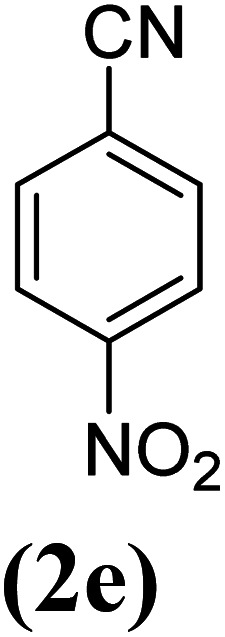	X = I, 86
			X = Br, 78
6	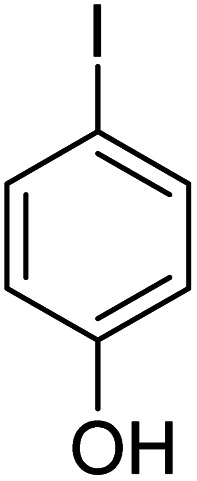	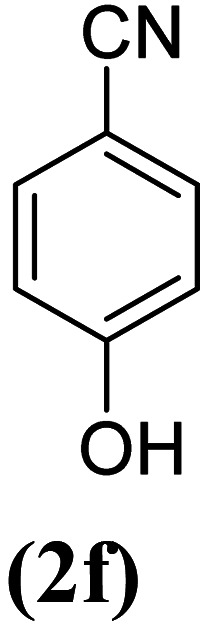	87
7	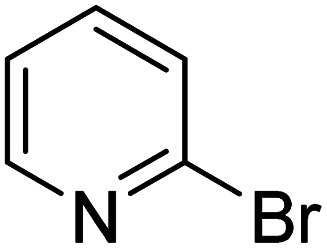	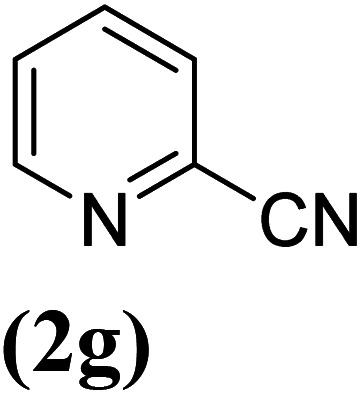	43
8	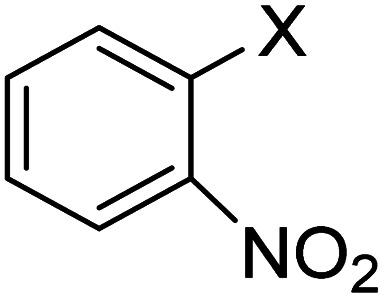	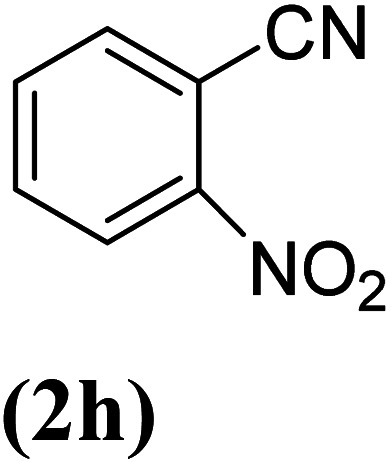	X = I, 78
			X = Br, 70
9	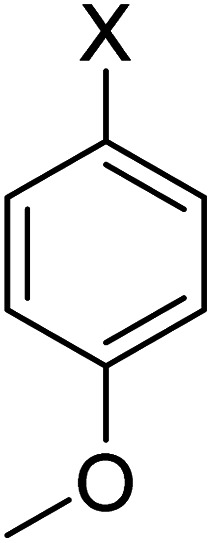	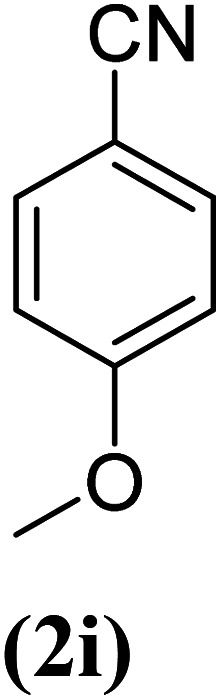	X = I, 94
			X = Br, 88

a Reaction conditions: 4-iodoanisole (1 mmol or 0.234 g), nitromethane (3 mmol or 0.183 g), potassium carbonate (1 mmol or 0.138 g), and the catalyst (30 mg), 12 h, 100 °C.

b Isolated yield.

The catalyst efficiency of Fe_3_O_4_@SiO_2_–TCT/B_5_–Cu(ii) and effectiveness of the present approach were compared with the previous researches. As shown in Table 3, it benefits from the use of copper as an inexpensive and readily available transition metal along with a copper-based heterogeneous catalyst, which can be easily separated from the reaction mixture. As an important and considerable advantage of this approach, it can be mentioned the use of nitromethane as an inexpensive, available, non-metallic CN source with low toxicity, which is highly efficient for producing desirable products in the presence of the Fe_3_O_4_@SiO_2_–TCT/B_5_–Cu(ii) catalyst.

**Table 3:** Comparison of the current approach with previous reported methods for benzonitrile synthesis

Entry	Catalyst	Condition	Yield^a^ (%)	Recyclability (run)	Reference
1	γ-Fe_2_O_3_–Pd–NHC–*n*-butyl-SO_3_Na	Aryl halide (1 mmol), K_4_[Fe(CN)_6_]·3H_2_O (1.2 mmol), Et_3_N (4 mmol), H_2_O (6 mL), catalyst (0.2 mol%)	94	6	31
2	CuI/Pd(ii)–AOFs	Aryl halide (10.0 mmol), potassium ferrocyanide (4.0 mmol), Cu(i)Pd(ii)-AOFs (6 mol%), Na_2_CO_3_(10.0 mmol), DMAC, reflux	98	4	32
3	Fe_3_O_4_@PMDP/Pd	Aryl halide (1.0 mmol), K_4_[Fe(CN)_6_] (0.17 mmol), Fe_3_O_4_@PMDP/Pd (1.5 mol%), Na_2_CO_3_ (1.5 mmol), DMF(3 mL), 120 °C	95	8	33
4	Pd NPs@β-CD	Aryl halide (1.5 mmol), potassium ferrocyanide (0.2 mmol), Na_2_CO_3_(1.8 mmol), catalyst (0.05 mol%), DMF, 120 °C	95	8	34
5	Ni(acac)_2_ (5 mol%), AlCl_3_ (10 mol%), bpy (30 mol%)	Aryl halide (0.2 mmol), Ni(acac)_2_ (5 mol%), AlCl_3_ (10 mol%), Bpy (30 mol%), ZnO(1.0 equiv.), HCONH_2_(1.0 mL), 1,2-DME (1.5 mL), 145 °C	90	—	35
6	CuF_2_	Aryl halide (0.2 mmol), CO(NH_2_)_2_ (4 equiv.), CuF_2_ (20 mol%), Li_2_CO_3_ (3 equiv.), O_2_, DMSO, 150 °C	87	—	36
7	Fe_3_O_4_@SiO_2_–TCT/B_5_–Cu(ii)	Aryl halide (1 mmol), CH_3_NO_2_ (3 mmol), K_2_CO_3_ (1 mmol), catalyst (30 mg), 100 °C		5	This work

a Isolated yield.

### 3.4. Mechanism

**Scheme 3:**
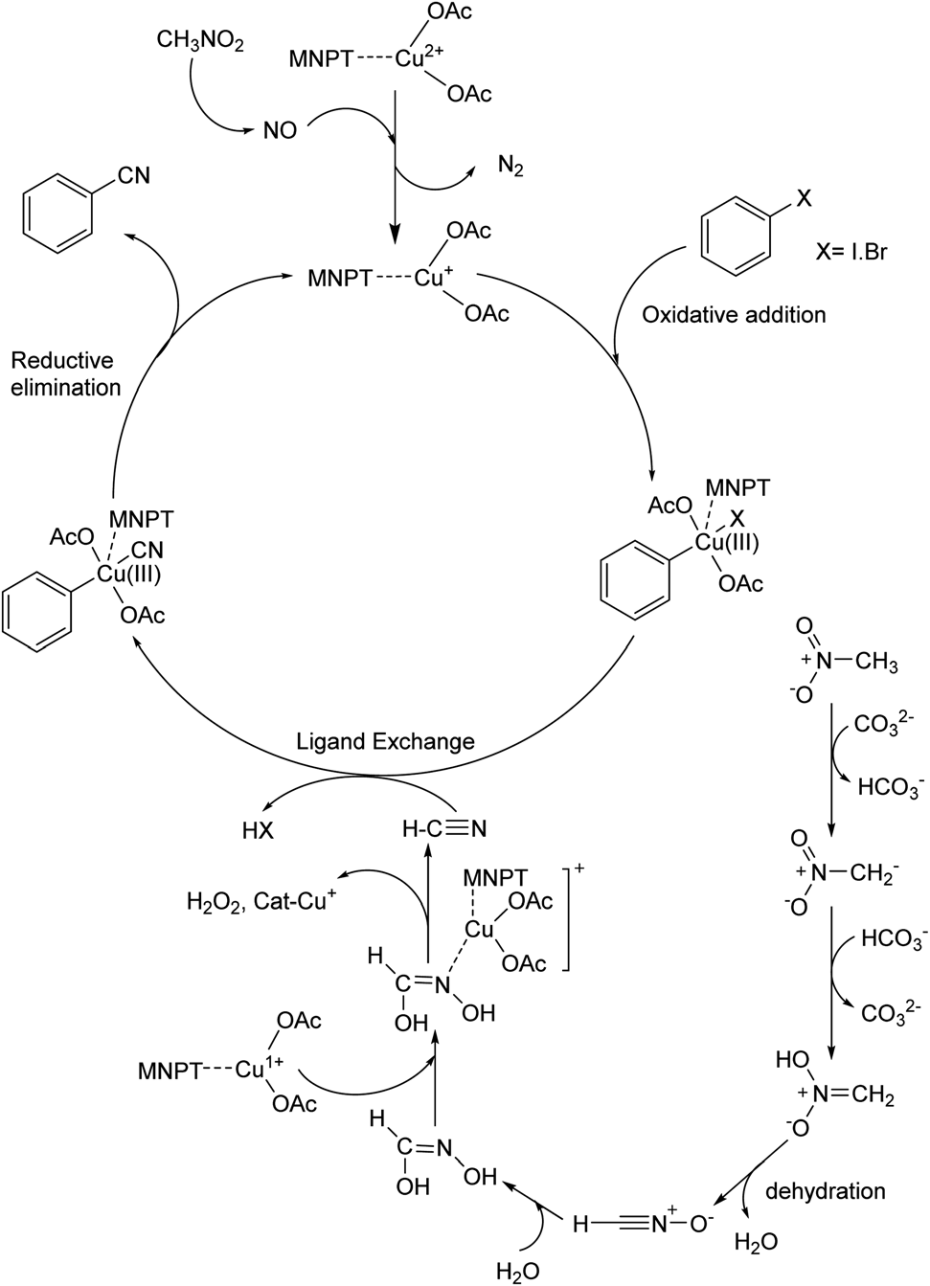
Plausible mechanism of cyanation reaction.

A possible mechanism for this reaction is proposed based on a similar previous report.^19*a*,37,38^ Thermochemical studies show that CH_3_NO_2_ can be decomposed into CO and NO at high temperatures. The released NO in the reaction medium interacts with Cu(ii) in the catalyst and reduces Cu(ii) to Cu(i). The 16-electron Cu(i) complex undergoes an oxidative addition reaction with aryl halide to form complex A. The 18-electron A complex performs ligand exchange in presence of HCN (generated from CH_3_NO_2_ using base) and produces B complex. Finally, complex B after a reductive elimination affords the cyanation product and the catalyst returns to the catalytic cycle (Scheme 3).

### 3.5. Catalyst recyclability

**Fig. 7:**
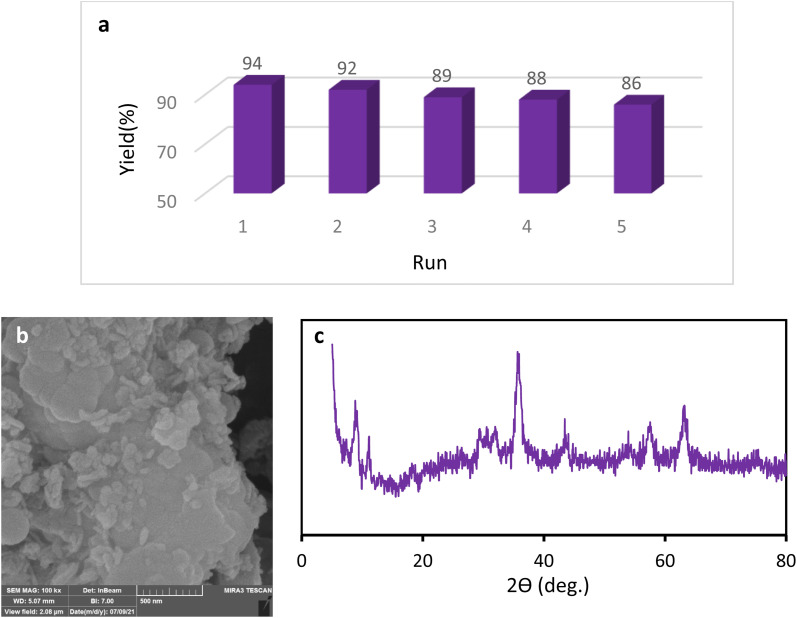
Recycling of the Fe_3_O_4_@SiO_2_–TCT/B_5_–Cu(ii) nanocatalyst (a), SEM image (b) and XRD pattern (c) of the recovered Fe_3_O_4_@SiO_2_–TCT/B_5_–Cu(ii) nanocatalyst.

We examined the recyclability and reusability of our catalyst in the cyanation reaction of 4-ome-iodobenzene in presence of CH_3_NO_2_ under the optimized conditions. The recyclability of the catalyst was evaluated for 5 consecutive runs. After each run of the reaction, catalyst was isolated by an external magnet, washed with EtOH and applied for the next run. As shown in Fig. 7, the catalytic activity has not changed significantly after 5 runs. The recycled catalyst in the 5th run was studied with SEM, XRD, and ICP analyses to investigate the catalyst stability under reaction conditions. The XRD pattern and SEM image have exhibited no significant difference with fresh catalyst. ICP analysis provided the Cu content of the recovered nanocatalyst after 5th run (0.65 mmol g^−1^) which did not show a significant decrease compared to that of the original catalyst.

## 4 Conclusion

In conclusion, a novel and efficient catalytic approach for the synthesis of aryl nitriles in presence of Fe_3_O_4_@SiO_2_–TCT/B_5_–Cu(ii) nanocatalyst and nitromethane as a non-toxic, inexpensive and readily available cyanating source has been developed. The aryl nitriles were obtained in moderate to good yields. The prepared heterogeneous nanocatalyst can be readily separated from the reaction mixture by magnetic filtration and recovered for at least five cycles without a significant loss of its activity.

## Ethical statement

This research does not involve human participants and/or animals.

## Data availability

The data that supports the findings of this study are available in the ESI† of this article.

## Author contributions

Conceptualization: Farzaneh Karimi, Masoumeh Jadidi Nejad; methodology: Farzaneh Karimi, Masoumeh Jadidi Nejad; formal analysis and investigation: Farzaneh Karimi and Masoumeh Jadidi Nejad; writing-original draft preparation: Masoumeh Jadidi Nejad, Arefe Salamatmanesh; writing-review and editing: Arefe Salamatmanesh, Masoumeh Jadidi Nejad; funding acquisition: Akbar Heydari; resources: Akbar Heydari; supervision: Akbar Heydari.

## Conflicts of interest

There are no conflicts of interest to declare.

## Supplementary Material

RA-013-D2RA06104J-s001
