# Supplementary material for: Magnetically separable triazine-based Cu(ii)–vitamin B5 complex in nitromethane toward efficient heterogeneous cyanation reaction of aryl halides
Source: RSC Adv. 2023 Jan 5;13(2):1412–21. doi: 10.1039/d2ra06104j (PMC9813807; doi:10.1039/d2ra06104j)
Supplement: RA-013-D2RA06104J-s001 [file RA-013-D2RA06104J-s001.pdf]

Electronic Supplementary Material (ESI) for RSC Advances.
This journal is © The Royal Society of Chemistry 2022

Magnetically separable triazine-based Cu(II)-vitamin B_5 complex in nitromethane toward efficient heterogeneous cyanation reaction of aryl halides

Farzaneh Karimi‡[a], Masoumeh Jadidi Nejad‡[a], Arefe Salamatmanesh‡[a] and Akbar Heydari*[a]

[a]*Chemistry Department, Tarbiat Modares University, P.O. Box 14155-4838, Tehran, Iran.*

**E-mail: heydar_a@modares.ac.ir; Fax: +98-21-82883455; Tel: +98-21-82883444.*

‡ These authors contributed equally.

Table of contents

**1. Characterization of aryl nitrile derivatives**	**S1**
**2. ^{1}H NMR TCT/B_5**	**S3**
**3. ^{1}H NMR and ^{13}CNMR spectra of aryl nitrile derivatives**	**S4**
**4. References**	**S20**

Characterization of aryl nitrile derivatives:

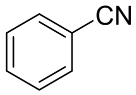

Benzonitrile (2a). Colorless oil; ^{1}H NMR (499 MHz, DMSO-d_6) δ 7.80 (d, J = 7.6 Hz, 2H), 7.71 – 7.68 (m, 1H), 7.55 (t, J = 7.6 Hz, 2H); ^{13}C NMR (126 MHz, DMSO-d6) δ 132.64, 131.17, 128.80, 118.60, 112.66. HRMS (ESI): Calcd for C_7H_5N (M +H) $^+$ 103.04, found 103.10.

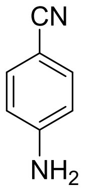

4-Aminobenzonitrile (2d). White solid, mp 83-85 ° C; ^{1}H NMR (499 MHz, DMSO-d_6) δ 7.36 (d, J = 8.1 Hz, 2H), 6.59 (d, J = 8.2 Hz, 2H), 6.11 (s, 2H); ^{13}C NMR (126 MHz, DMSO-d6) δ 150.98, 133.14, 118.42, 114.65, 98.61. HRMS (ESI): Calcd for $C_7H_6N_2$ (M +H) $^+$ 118.05, found 118.10.

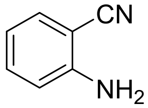
2-Aminobenzonitrile (2f). Beige-brown solid, mp 49-50 ^{o} C; ^{1}H NMR (499 MHz, DMSO-d_6) δ 7.35 (d, *J* = 7.9 Hz, 1H), 7.28 (t, *J* = 7.5 Hz, 1H), 6.76 (d, *J* = 8.5 Hz, 1H), 6.57 (t, *J* = 7.5 Hz, 1H), 5.98 (s, 2H); ^{13}C NMR (126 MHz, DMSO-d_6) δ 151.11, 133.59, 13.94, 118.02, 116.74, 94.30. 115.05. HRMS (ESI): Calcd for $C_7H_6N_2$ (M +H) $^{+}$ 118.05, found 118.10.

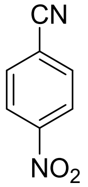
4-Nitrobenzonitrile (2h). Yellow solid, mp 147-149 ^{o} C; ^{1}H NMR (499 MHz, DMSO-d_6) δ 8.37 (d, *J* = 8.3 Hz, 2H), 8.16 (d, *J* = 8.3 Hz, 2H); ^{13}C NMR (126 MHz, DMSO-d_6) δ 15.01, 133.80, 123.92, 117.93, 117.07. HRMS (ESI): Calcd for $C_7H_4N_2O_2$ (M +H) $^{+}$ 148.03, found 148.10.

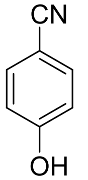
4-Hydroxybenzonitrile (2i). White solid, mp 110-112 ^{o} C; ^{1}H NMR (499 MHz, DMSO-d_6) δ 10.59 (s, 1H), 7.59 (d, *J* = 8.2 Hz, 2H), 6.88 (d, *J* = 8.3 Hz, 2H); ^{13}C NMR (126 MHz, DMSO-d_6) δ 161.81, 134.20, 118.55, 116.26, 102.34. HRMS (ESI): Calcd for C_7H_5NO (M +H) $^{+}$ 119.04, found 119.20.

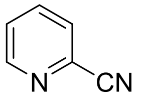
Picolinonitrile (2j). White solid, mp 28-30 ^{o} C; ^{1}H NMR (499 MHz, DMSO-d_6) δ 8.75 (d, *J* = 4.8 Hz, 1H), 8.07 – 8.01 (m, 2H), 7.73 (t, *J* = 6.1 Hz, 1H); ^{13}C NMR (126 MHz, DMSO-d_6) δ 151.37, 138.00, 133.98, 128.20, 127.66,117.32. HRMS (ESI): Calcd for $C_6H_4N_2$ (M +H) $^{+}$ 104.04, found 104.10.

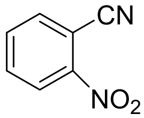
2-Nitrobenzonitrile (2k). Yellow solid, mp 109-110 ^{o} C; ^{1}H NMR (499 MHz, DMSO-d_6) δ 8.42 – 8.35 (m, 1H), 8.17 (dd, *J* = 5.7, 3.4 Hz, 1H), 7.98 (dd, *J* = 6.0, 3.4 Hz, 2H); ^{13}C NMR (126 MHz, DMSO-d_6) δ 149.39, 135.51, 134.36, 133.83, 124.14, 115.13,107.96. HRMS (ESI): Calcd for $C_7H_4N_2O_2$ (M +H) $^{+}$ 148.03, found 148.10.

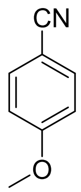

4-Methoxybenzonitrile (2m). White solid, mp 60-61 ° C; ^{1}H NMR (499 MHz, DMSO-d_6) δ 7.76 (d, J = 8.6 Hz, 2H), 7.09 (d, J = 8.6 Hz, 2H), 3.83 (s, 3H); ^{13}C NMR (126 MHz, DMSO-d_6) δ 163.10, 133.88, 118.55, 114.22, 104.08, 55.32.. HRMS (ESI): Calcd for $C_{13}H_{13}N_2O$ $(M+H)^+$ 133.05, found 133.10.

^{1}H NMR of TCT/B_5

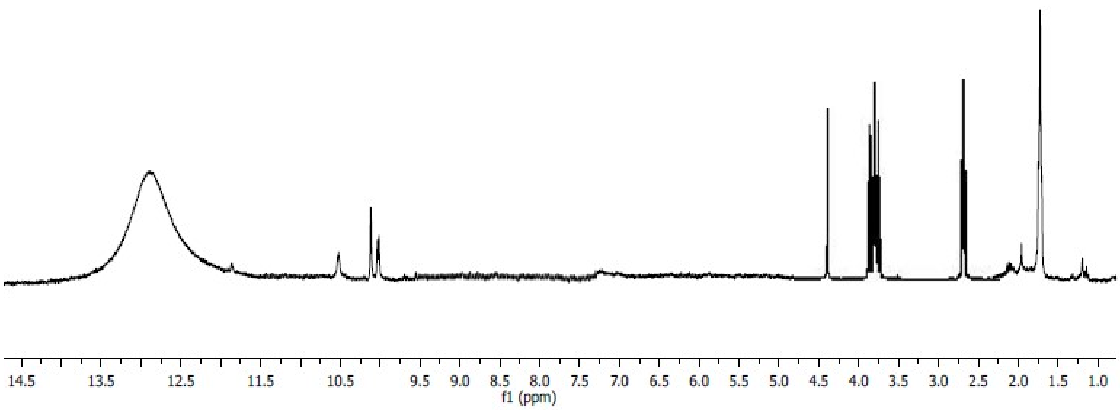

^{1}H NMR and ^{13}C NMR spectra of aryl nitrile derivatives:

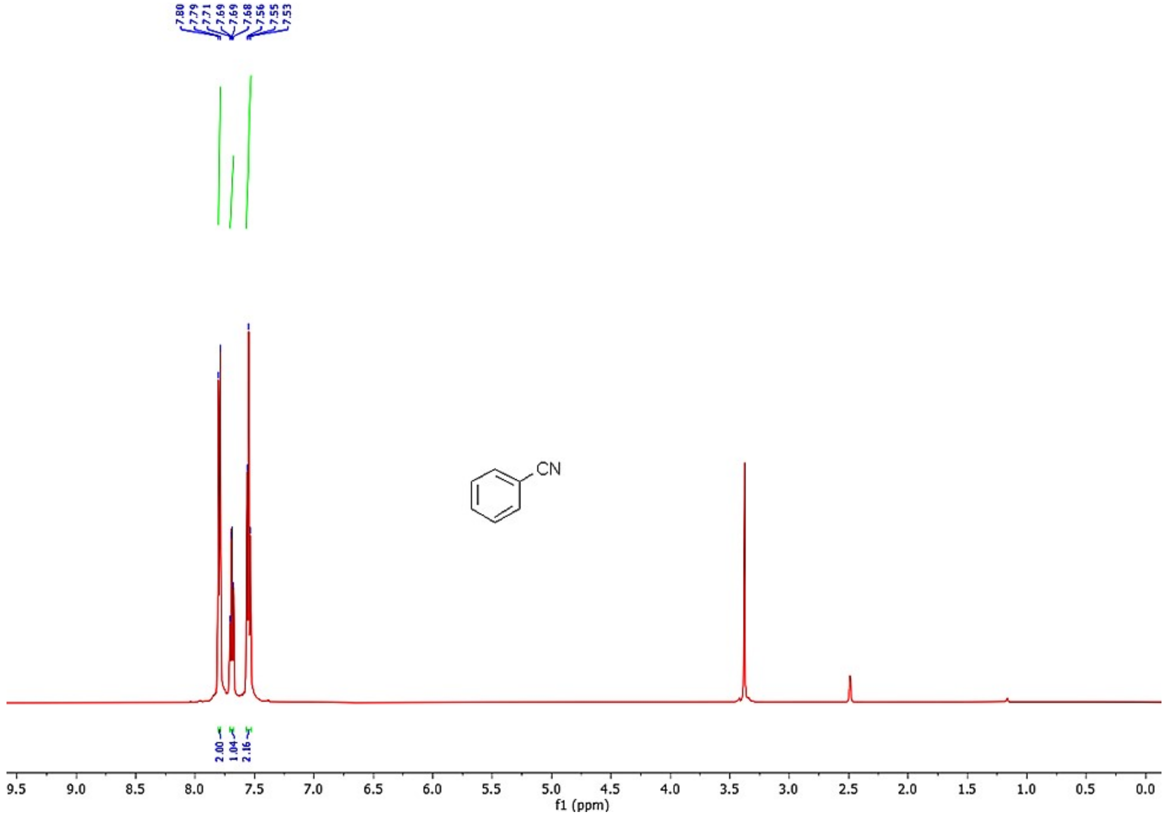

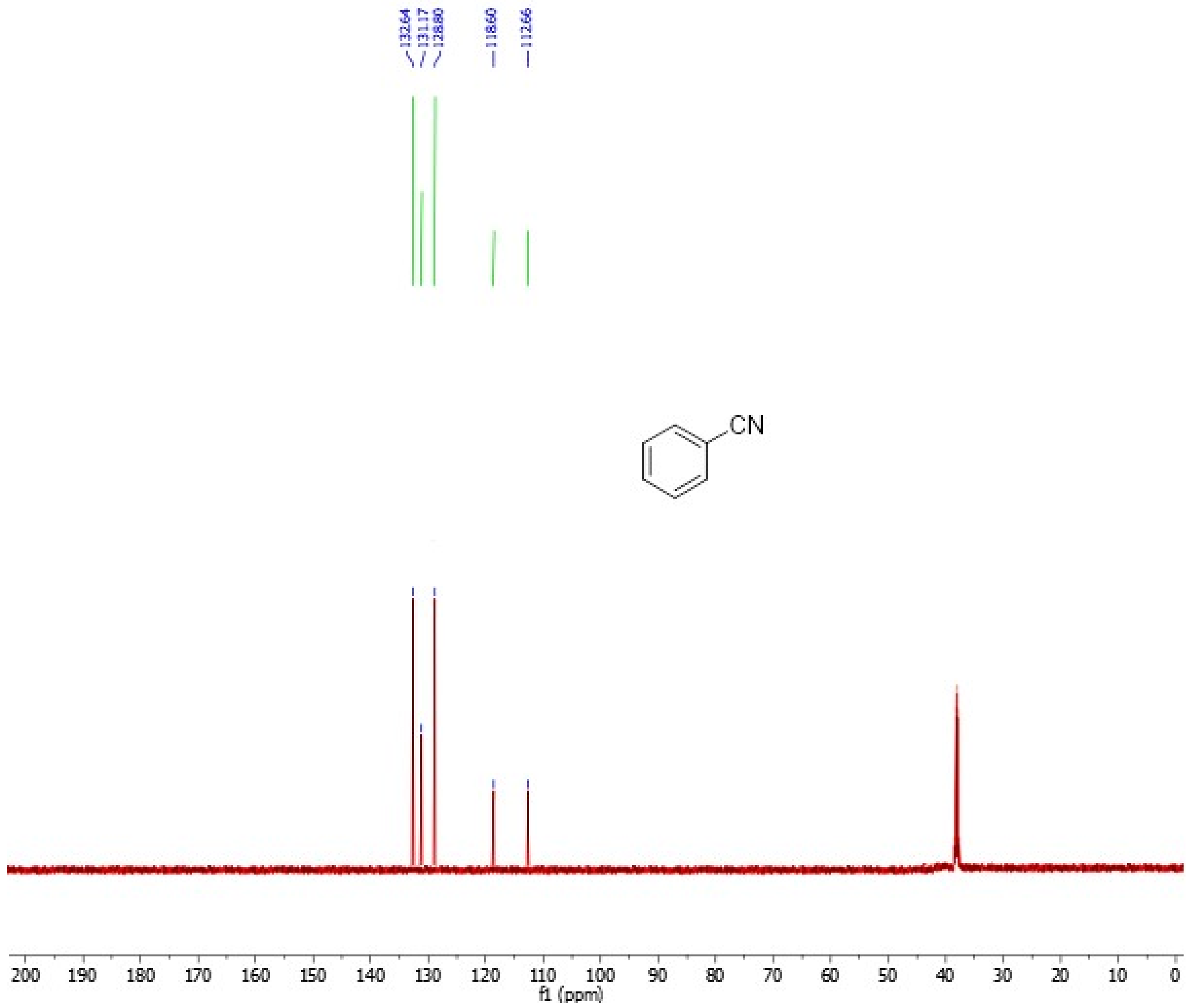

132.64
131.17
128.80
118.60
112.66
CN
200
190
180
170
160
150
140
130
120
110
100
90
80
70
60
50
40
30
20
10
0
f1 (ppm)

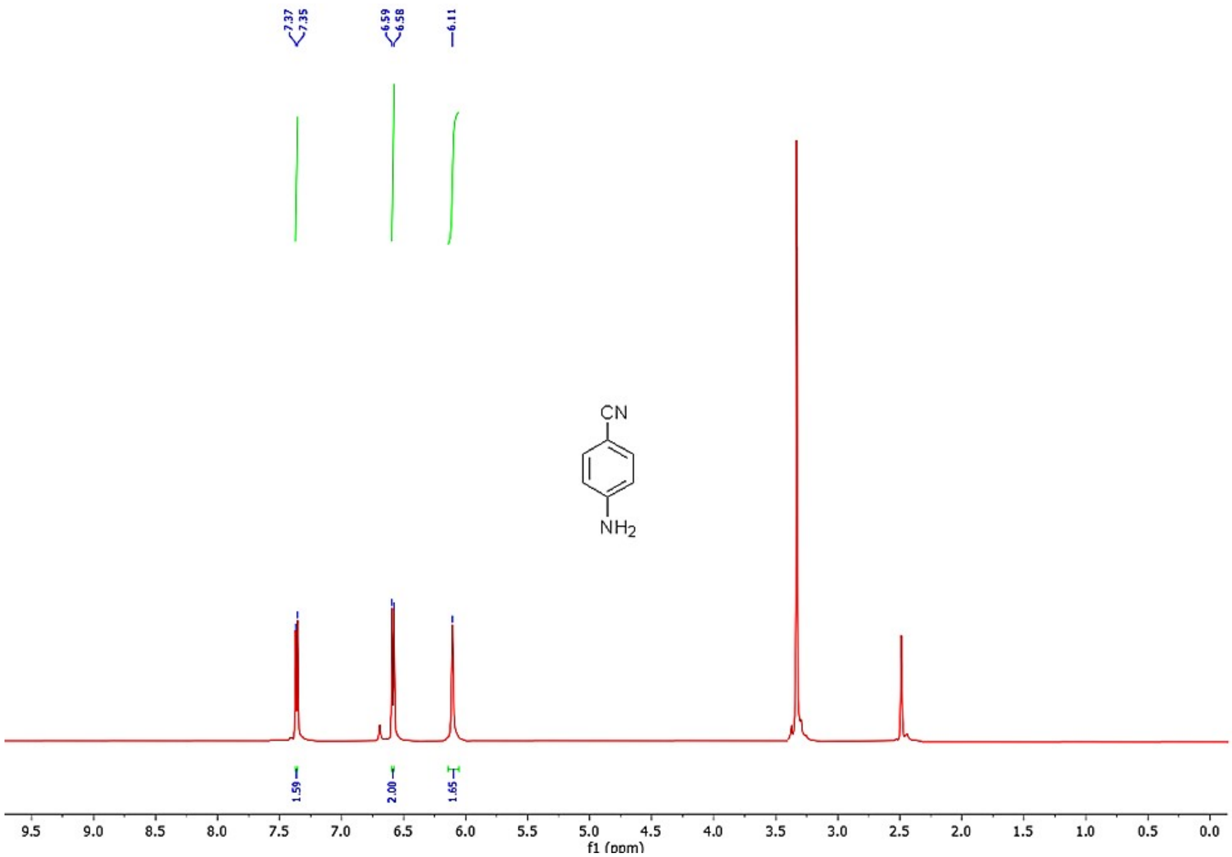
7.37
7.35
6.59
6.58
6.11
CN
NH2
1.59
2.00
1.65
f1 (ppm)

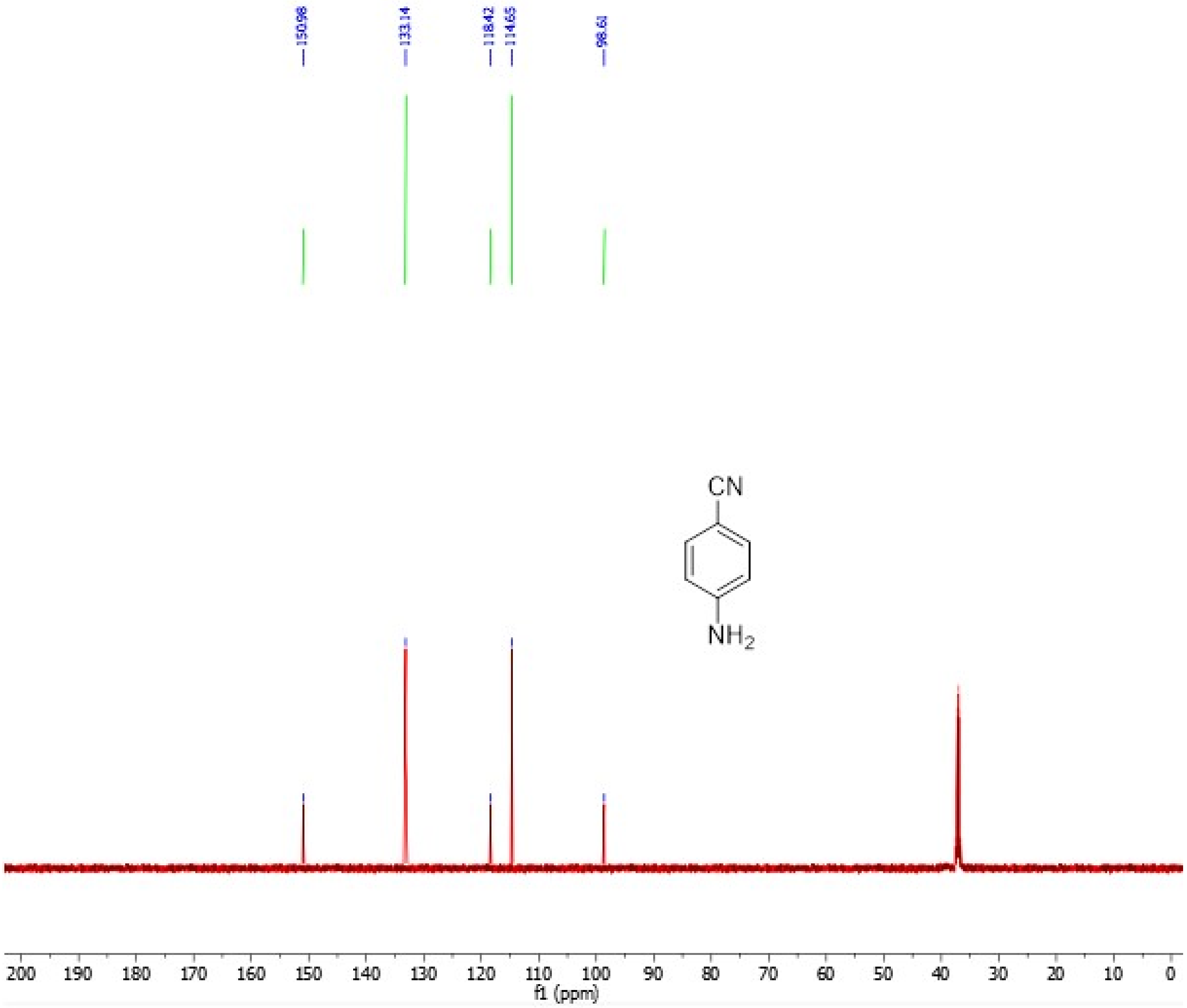

150.98
133.14
118.42
114.65
98.61
CN
NH2
200
190
180
170
160
150
140
130
120
110
100
90
80
70
60
50
40
30
20
10
0
f1 (ppm)

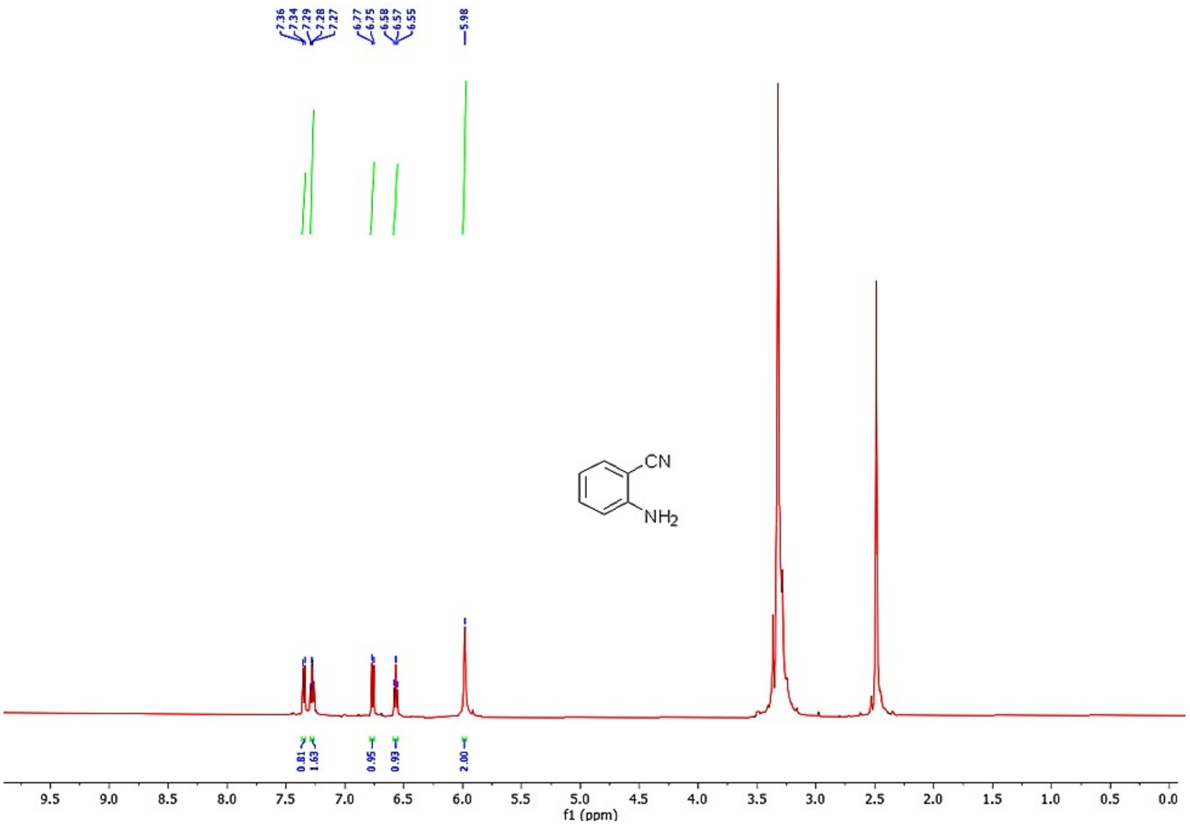
7.36
7.34
7.29
7.28
7.27
6.77
6.75
6.58
6.57
6.55
5.98
CN
NH2
0.81
1.63
0.95
0.93
2.00
9.5
9.0
8.5
8.0
7.5
7.0
6.5
6.0
5.5
5.0
4.5
4.0
3.5
3.0
2.5
2.0
1.5
1.0
0.5
0.0
f1 (ppm)

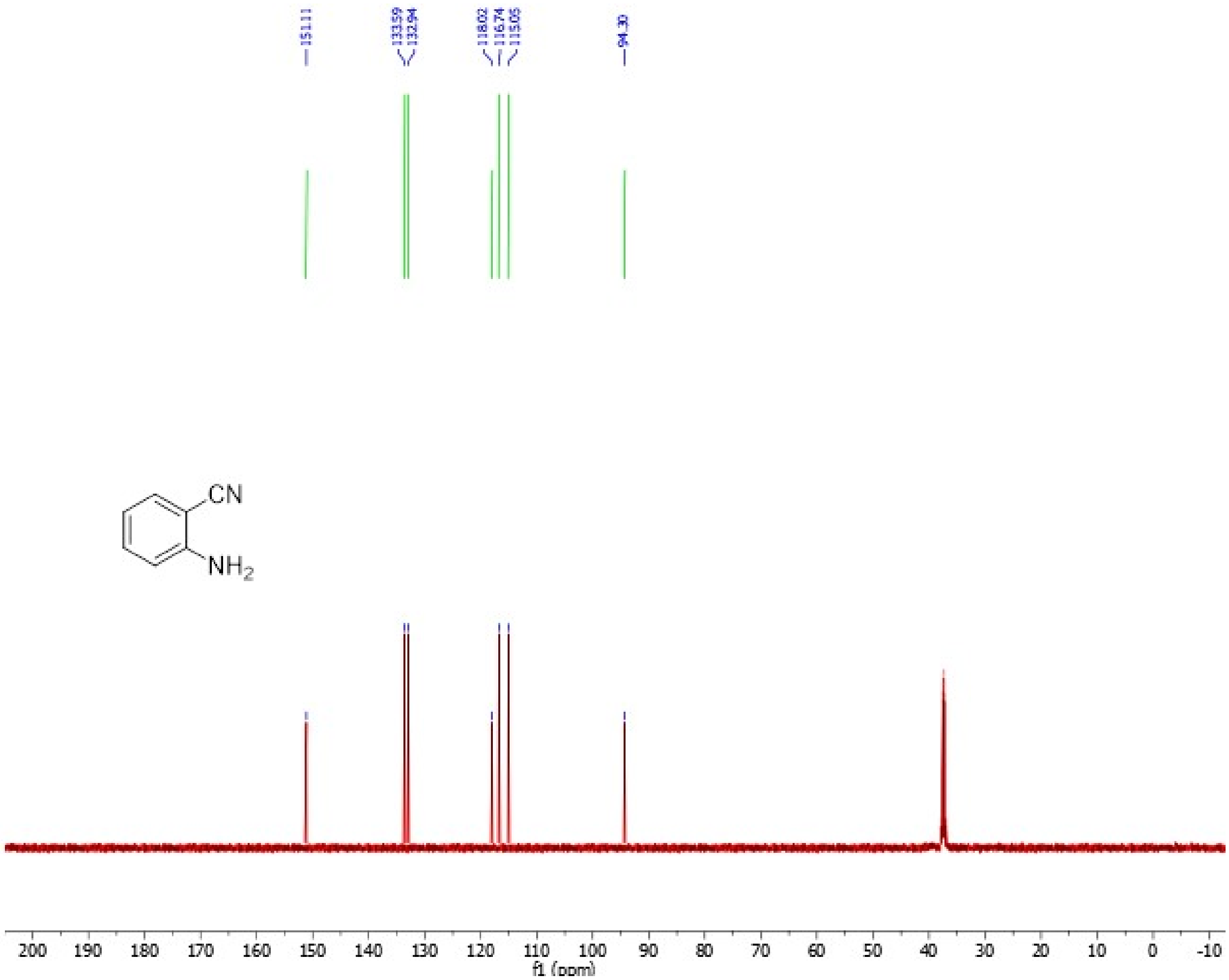

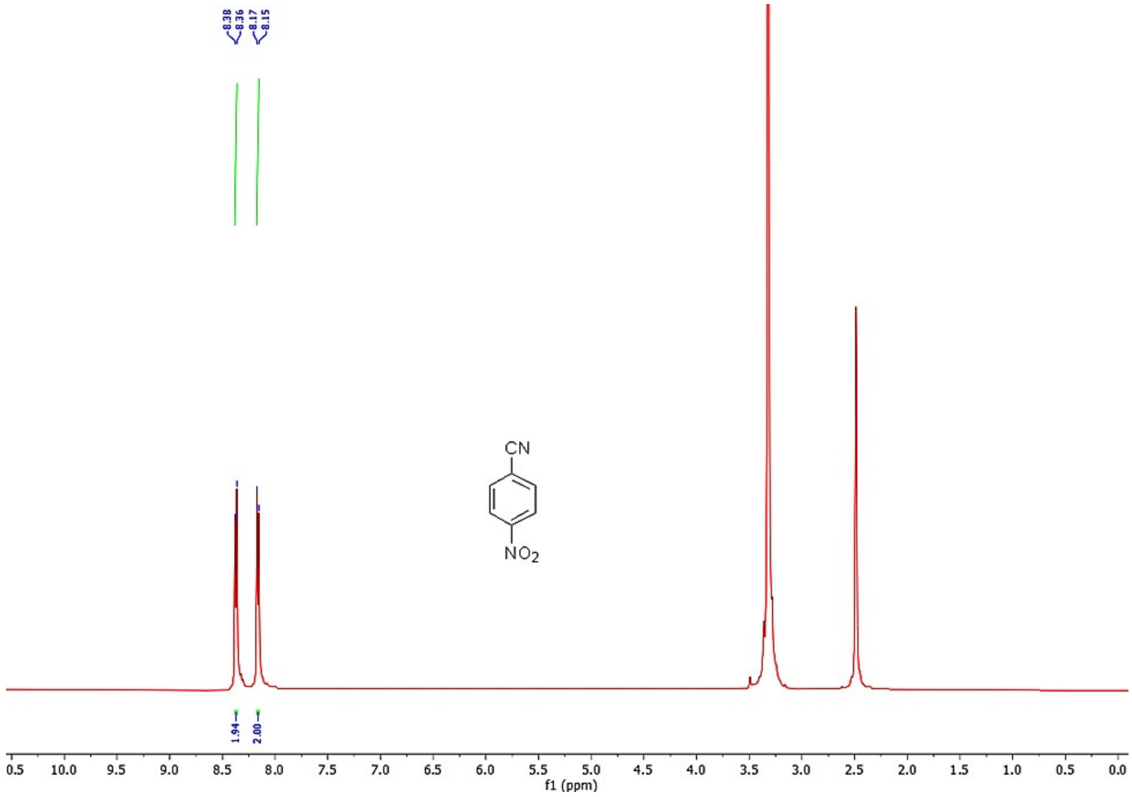
8.38
8.36
8.17
8.15
CN
NO2
1.94
2.00
0.5
10.0
9.5
9.0
8.5
8.0
7.5
7.0
6.5
6.0
5.5
5.0
4.5
4.0
3.5
3.0
2.5
2.0
1.5
1.0
0.5
0.0
f1 (ppm)

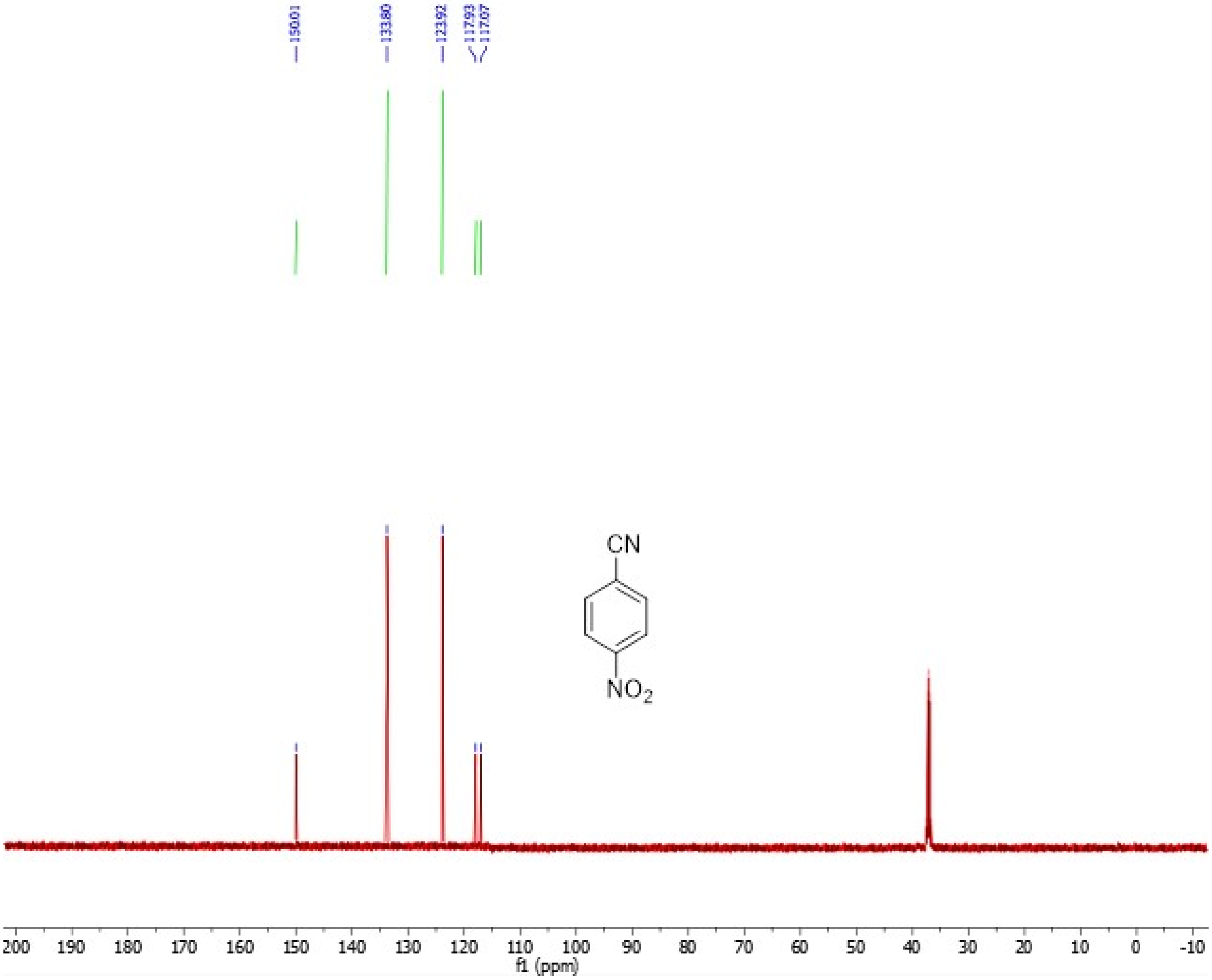

150.01
133.80
123.92
117.93
117.07
CN
NO2
200 190 180 170 160 150 140 130 120 110 100 90 80 70 60 50 40 30 20 10 0 -10
f1 (ppm)

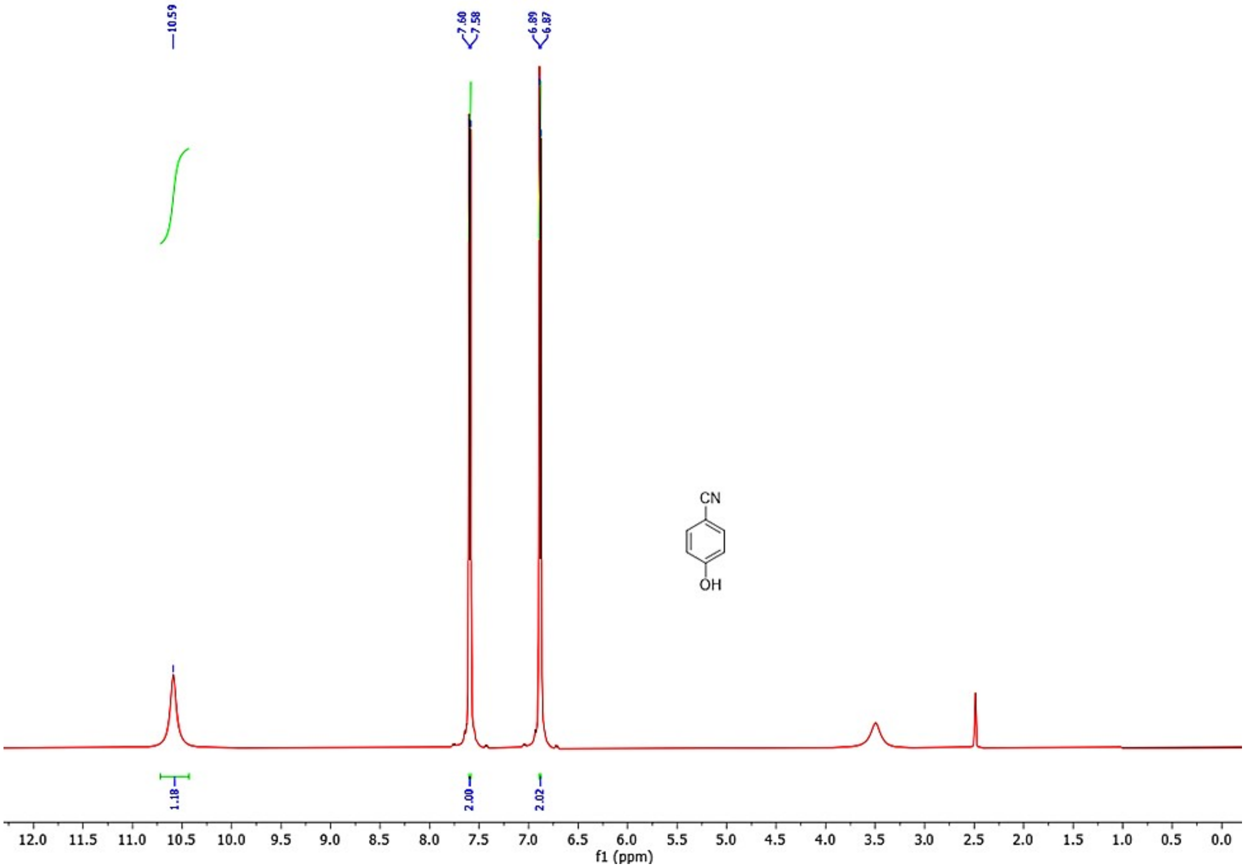

10.59
7.60
7.58
6.89
6.87
CN
OH
1.18
2.00
2.02
12.0 11.5 11.0 10.5 10.0 9.5 9.0 8.5 8.0 7.5 7.0 6.5 6.0 5.5 5.0 4.5 4.0 3.5 3.0 2.5 2.0 1.5 1.0 0.5 0.0
f1 (ppm)

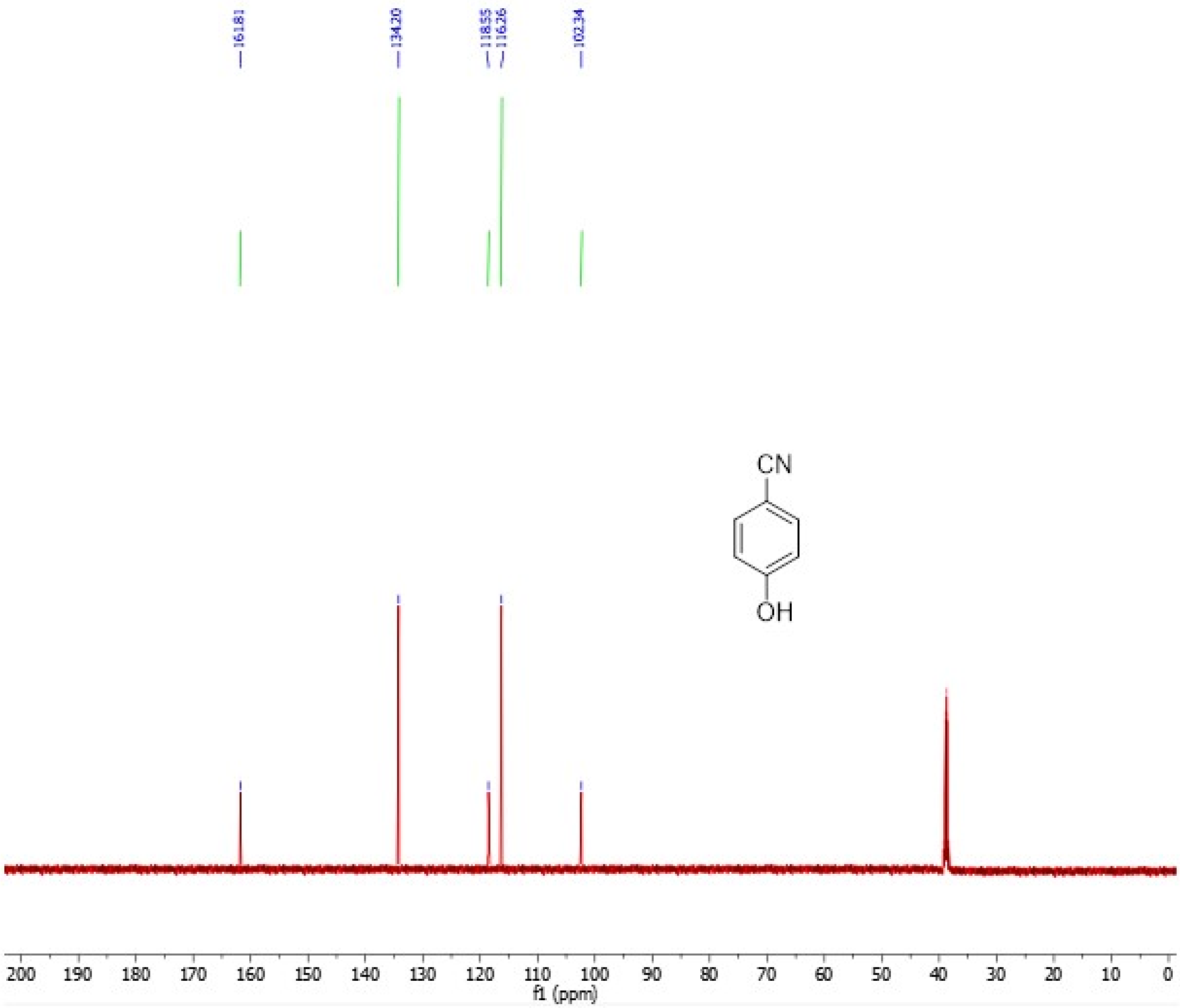
161.81
134.20
118.55
116.26
102.34
CN
OH
200
190
180
170
160
150
140
130
120
110
100
90
80
70
60
50
40
30
20
10
0
f1 (ppm)

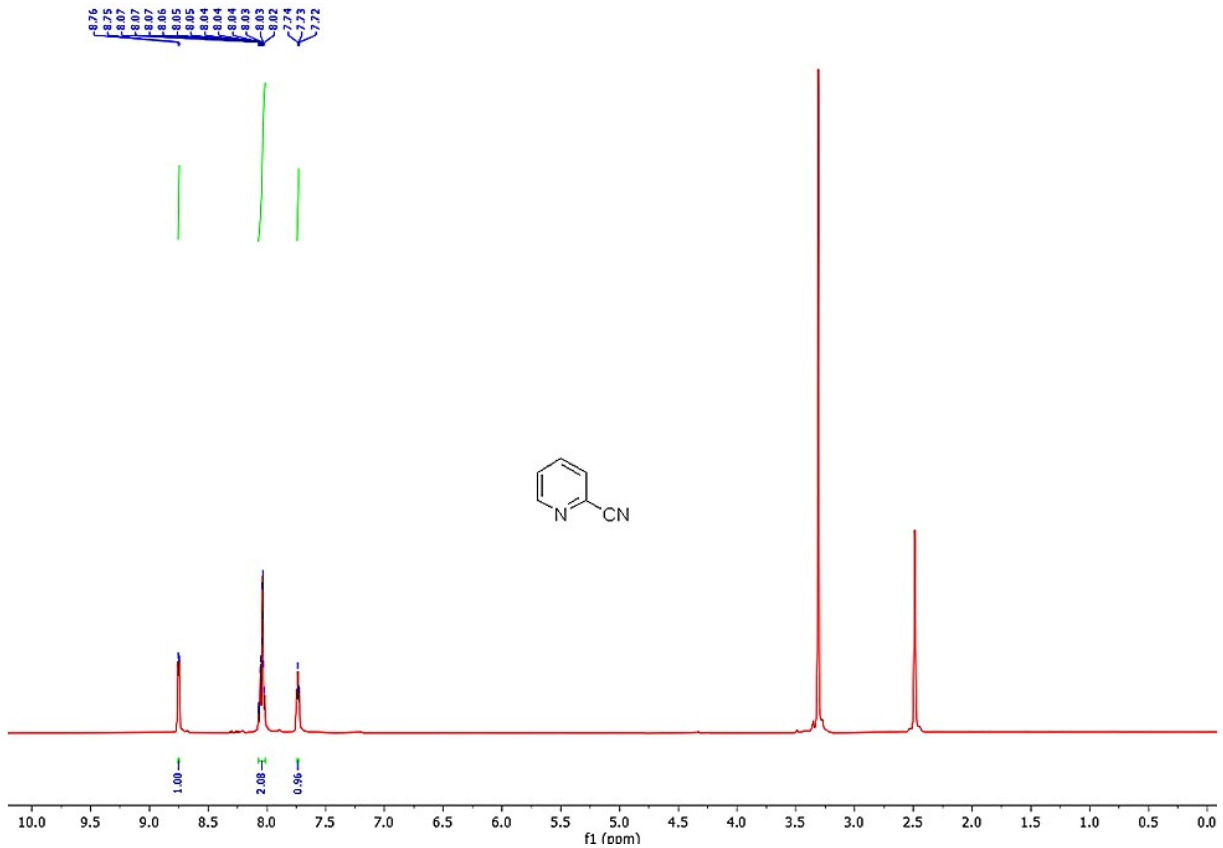
8.76
8.75
8.07
8.07
8.07
8.06
8.05
8.05
8.04
8.04
8.04
8.03
8.03
8.02
7.74
7.73
7.72
N
CN
1.00
2.08
0.96
10.0 9.5 9.0 8.5 8.0 7.5 7.0 6.5 6.0 5.5 5.0 4.5 4.0 3.5 3.0 2.5 2.0 1.5 1.0 0.5 0.0
f1 (ppm)

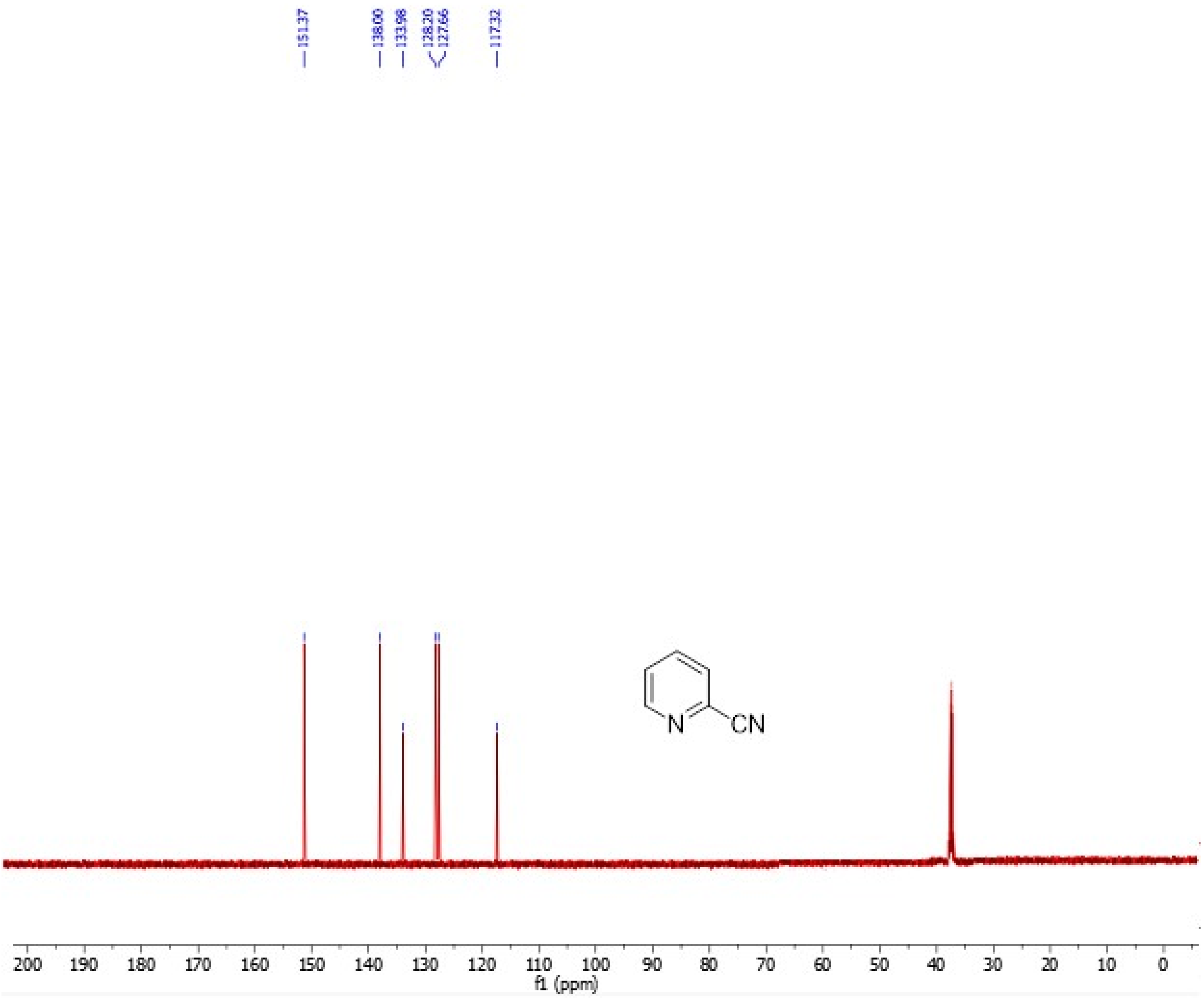

151.37
138.00
133.98
128.20
127.66
117.32
CN
N
200 190 180 170 160 150 140 130 120 110 100 90 80 70 60 50 40 30 20 10 0
f1 (ppm)

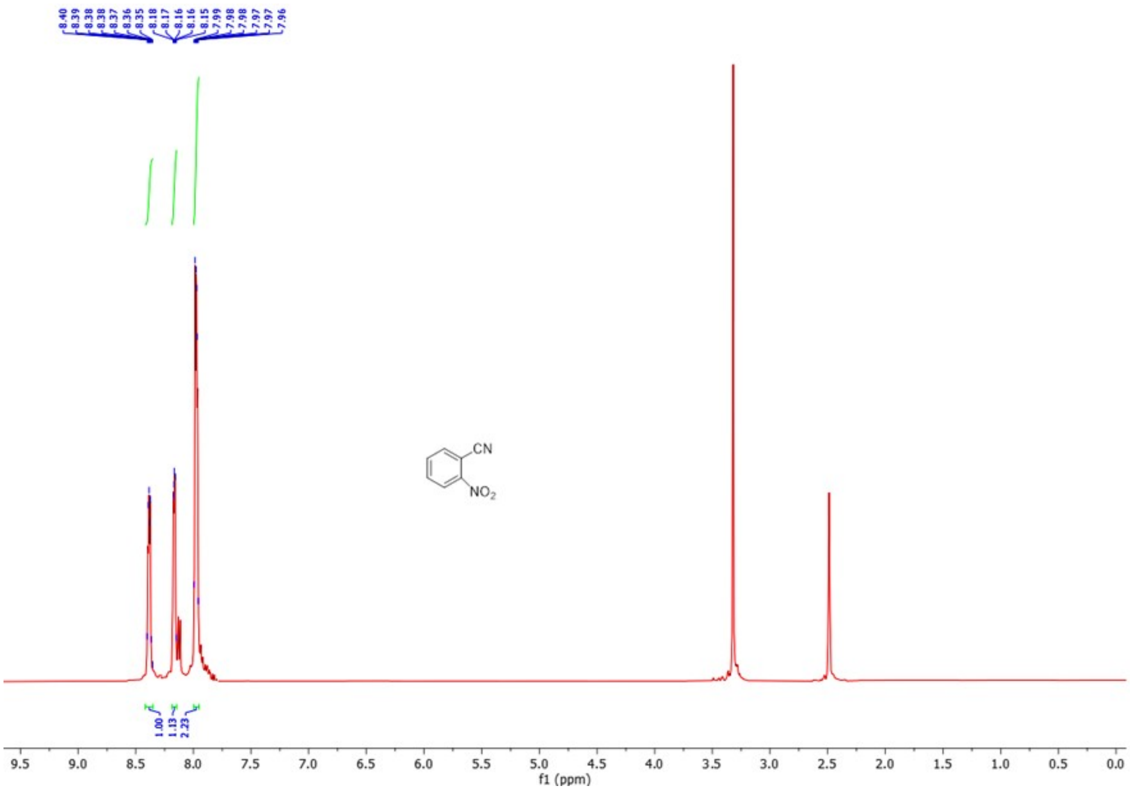

8.40
8.39
8.38
8.38
8.37
8.36
8.35
8.18
8.17
8.16
8.16
8.15
7.99
7.98
7.98
7.97
7.97
7.96
CN
NO2
1.00
1.13
2.23
9.5
9.0
8.5
8.0
7.5
7.0
6.5
6.0
5.5
5.0
4.5
4.0
3.5
3.0
2.5
2.0
1.5
1.0
0.5
0.0
f1 (ppm)

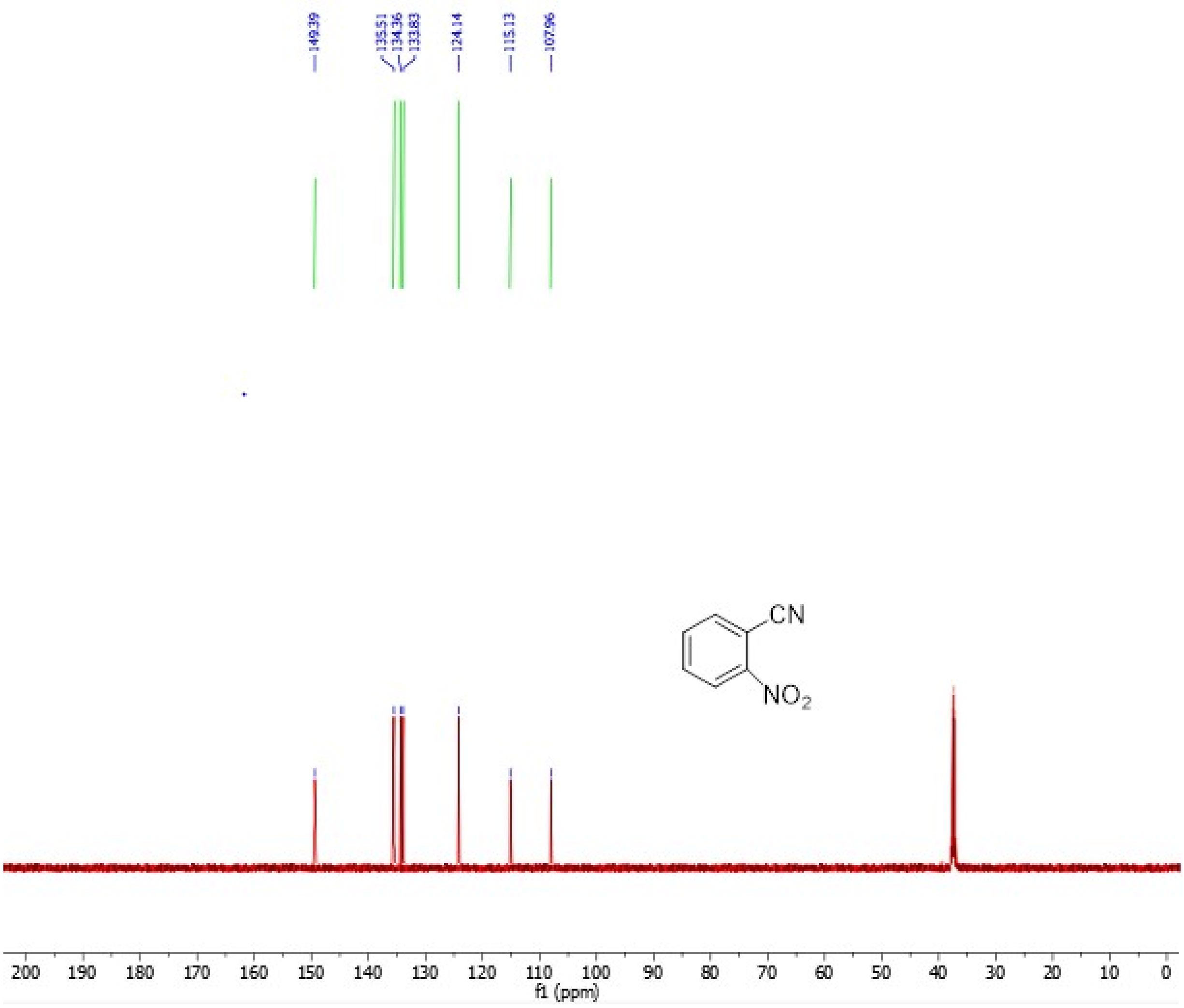
149.39
135.51
134.36
133.83
124.14
115.13
107.96
CN
NO2
200
190
180
170
160
150
140
130
120
110
100
90
80
70
60
50
40
30
20
10
0
f1 (ppm)

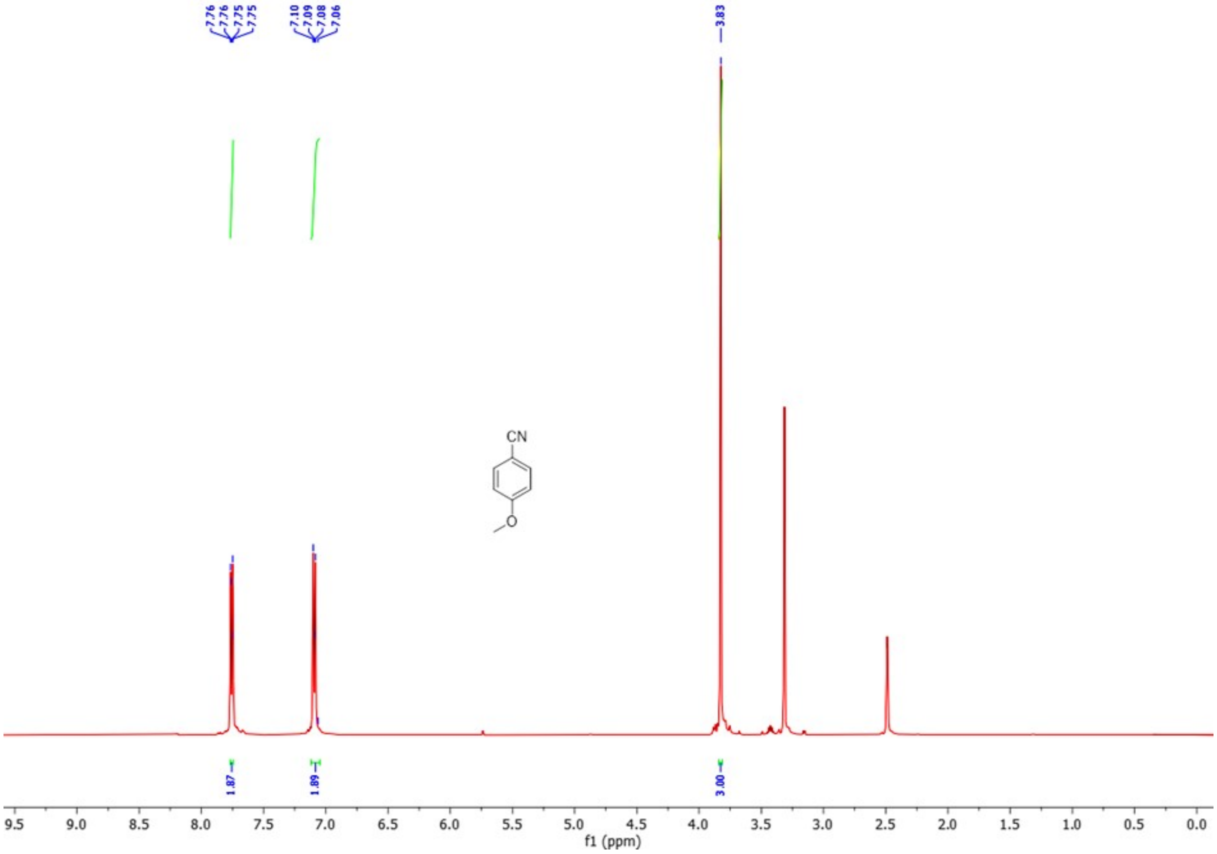

7.76
7.76
7.75
7.75
7.10
7.09
7.08
7.06
3.83
CN
O
1.87
1.89
3.00
9.5
9.0
8.5
8.0
7.5
7.0
6.5
6.0
5.5
5.0
4.5
4.0
3.5
3.0
2.5
2.0
1.5
1.0
0.5
0.0
f1 (ppm)

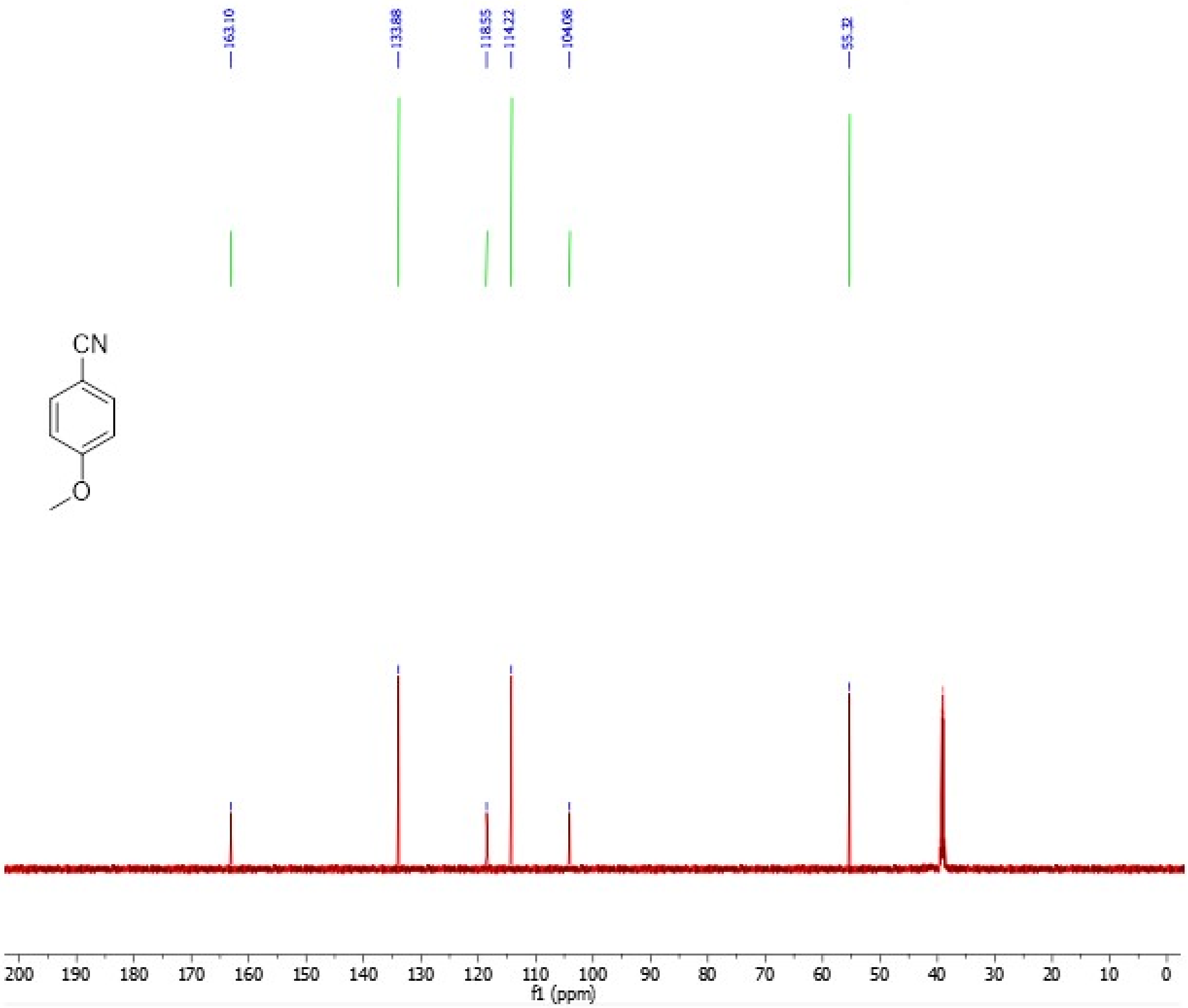
163.10
133.88
118.55
114.22
104.08
55.32
CN
O
200
190
180
170
160
150
140
130
120
110
100
90
80
70
60
50
40
30
20
10
0
f1 (ppm)

References:

Han, Min Su; Jang, Mingyeong; Lim, Taeho; Park, Byoung Yong, Journal of Organic Chemistry, 2022, 87, 910 – 919.

Xu, Da Peng; Xiong, Meilu; Kazem nejadi, Milad, RSC Advances, 2021, 11, 21, 12484 – 12499.

Dighe, Shashikant U.; Chowdhury, Deepan; Batra, Sanjay, Advanced Synthesis and Catalysis, 2014, 356, 3892 – 3896.

Shimojo, Hiroyuki; Moriyama, Katsuhiko; Togo, Hideo, Synthesis, 2013, 45, 2164.
